# Evolution and Functional Differentiation of the C-terminal Motifs of FtsZs During Plant Evolution

**DOI:** 10.1093/molbev/msae145

**Published:** 2024-07-15

**Authors:** Jinjie An, Lulu Wang, Conghao Hong, Hongbo Gao

**Affiliations:** National Engineering Research Center of Tree Breeding and Ecological Restoration, State Key Laboratory of Efficient Production of Forest Resources, College of Biological Sciences and Technology, Beijing Forestry University, Beijing 100083, China; National Engineering Research Center of Tree Breeding and Ecological Restoration, State Key Laboratory of Efficient Production of Forest Resources, College of Biological Sciences and Technology, Beijing Forestry University, Beijing 100083, China; National Engineering Research Center of Tree Breeding and Ecological Restoration, State Key Laboratory of Efficient Production of Forest Resources, College of Biological Sciences and Technology, Beijing Forestry University, Beijing 100083, China; National Engineering Research Center of Tree Breeding and Ecological Restoration, State Key Laboratory of Efficient Production of Forest Resources, College of Biological Sciences and Technology, Beijing Forestry University, Beijing 100083, China

**Keywords:** chloroplast division, FtsZ1, FtsZ2, C-terminal motif, evolution

## Abstract

Filamentous temperature-sensitive Z (FtsZ) is a tubulin-like GTPase that is highly conserved in bacteria and plants. It polymerizes into a ring at the division site of bacteria and chloroplasts and serves as the scaffold protein of the division complex. While a single FtsZ is present in bacteria and cyanobacteria, there are two subfamilies, FtsZ1 and FtsZ2 in the green lineage, and FtsZA and FtsZB in red algae. In *Arabidopsis thaliana*, the C-terminal motifs of AtFtsZ1 (Z1C) and AtFtsZ2-1 (Z2C) display distinct functions in the regulation of chloroplast division. Z1C exhibits weak membrane-binding activity, whereas Z2C engages in the interaction with the membrane protein AtARC6. Here, we provide evidence revealing the distinct traits of the C-terminal motifs of FtsZ1 and FtsZ2 throughout the plant evolutionary process. In a range of plant species, the C-terminal motifs of FtsZ1 exhibit diverse membrane-binding properties critical for regulating chloroplast division. In chlorophytes, the C-terminal motifs of FtsZ1 and FtsZ2 exhibit both membrane-binding and protein interaction functions, which are similar to those of cyanobacterial FtsZ and red algal FtsZA. During the transition from algae to land plants, the functions of the C-terminal motifs of FtsZ1 and FtsZ2 exhibit differentiation. FtsZ1 lost the function of interacting with ARC6 in land plants, and the membrane-binding activity of FtsZ2 was lost in ferns. Our findings reveal the functional differentiation of the C-terminal motifs of FtsZs during plant evolution, which is critical for chloroplast division.

## Introduction

Chloroplasts originated from endosymbiotic events that occurred ∼1 billion years ago, leading to the emergence of the Archaeplastida clade, which includes Glaucophytes, Rhodophytes (red algae), and Chlorophytes (green algae) (Osteryoung and Nunnari 2003; Zimorski et al. 2014). Chlorophytes subsequently diverged into two major lineages: Chlorophytes and Charophytes, paving the way for the evolution of various terrestrial plant groups (de Vries et al. 2016; Wang et al. 2020). During the evolution of plants, land plants have evolved into different species ranging from simple to complex, such as bryophytes, lycophytes, ferns, gymnosperms, and angiosperms (Wickett et al. 2014; Szövényi et al. 2019).

Filamentous temperature-sensitive Z (FtsZ) is a tubulin-like cytoskeletal GTPase that plays an essential role in the division of bacteria and chloroplasts (Osteryoung and Nunnari 2003; Stokes and Osteryoung 2003). *FtsZ* genes also exist in some non-plant eukaryotes, such as ameba, excavate, and stramenopiles, and some of those genes may be involved in mitochondrial division (Beech et al. 2000; Leger et al. 2015). In both bacteria and chloroplasts, FtsZ polymerizes to form a contractile ring-like (Z ring) complex at the division site (Vitha et al. 2001; Errington et al. 2003). The polymerization of FtsZ activates GTPase activity, catalyzing the hydrolysis of GTP to generate contractile force (Scheffers et al. 2002; Huecas et al. 2007; Olson et al. 2010). The Z ring provides a scaffold for the division apparatus and recruits other protein components (Adams and Errington 2009; Osteryoung and Pyke 2014; Chen et al. 2018). The formation of the Z ring represents the beginning of division, and Z ring localization is regulated by the Min system proteins (de Boer et al. 1989; Fujiwara et al. 2008; Zhang et al. 2013; Shaik et al. 2018; Sun et al. 2023). Incorrect localization of the Z ring causes abnormal division of bacteria and chloroplasts (Vitha et al. 2001; Adams and Errington 2009; McQuillen and Xiao 2020).

In bacteria, including cyanobacteria, the FtsZ exists as a single form, which then diverges into FtsZ1 and FtsZ2 subfamilies in plants (green algae and land plants), and FtsZA and FtsZB in red algae (Stokes and Osteryoung 2003; TerBush et al. 2013; Chen et al. 2017). In *Physcomitrium patens*, there is a special FtsZ, FtsZ3, besides the four FtsZs of FtsZ1 and FtsZ2 subfamilies (Martin et al. 2009a,b). The co-polymerization of FtsZ1 and FtsZ2 at the division site is vital for chloroplast division in plants (McAndrew et al. 2001). Similar to FtsZ1 and FtsZ2, FtsZA and FtsZB can also co-polymerize to form heteropolymers (Chen et al. 2017). Notably, FtsZ2 and FtsZA share a resemblance with bacterial FtsZ, including a conserved C-terminal motif (Osteryoung and McAndrew 2001; TerBush et al. 2013, 2018). The FtsZ C-terminal motif of cyanobacteria interacts with membrane protein ZipN or Ftn2 (Vitha et al. 2003; Mazouni et al. 2004). This motif in FtsZ2 interacts with the membrane protein Accumulation and Replication of Chloroplast6 (ARC6), anchoring the Z ring to the chloroplast inner envelope membrane (Maple et al. 2005; Zhang et al. 2016; Wang et al. 2017). In red algae, ARC6 may promote FtsZ filament formation by interacting with FtsZA (Yoshida 2018). In contrast, FtsZB lack C-terminal domain, and FtsZ1 does not interact with any examined membrane protein (Miyagishima et al. 2004; Maple et al. 2005; Zhang et al. 2016).

Our previous research revealed that the C-terminal motif of AtFtsZ1 in *Arabidopsis thaliana* comprises an amphiphilic beta-strand, exhibiting weak membrane-binding capabilities (Liu et al. 2022). The absence of this motif in *Arabidopsis* perturbs FtsZ assembly, leading to aberrant chloroplast division. When expressed individually in *Escherichia coli*, AtFtsZ2-1 formed into straight filaments. However, fusion protein expression of AtFtsZ2-1 with AtFtsZ1 C10 or co-expression with AtFtsZ1 resulted in the formation of helical structures, likely attributable to the membrane-binding property of the Z1C motif.

In this study, we found that the C-terminus of FtsZ1 proteins is composed of a simple and variable motif in different species, which is beneficial for the function of the proteins during evolution. Employing an *E. coli* expression system (Irieda and Shiomi 2017), we analyzed the membrane-binding activities of different FtsZ1 C-terminal motifs, including those from chlorophytes, charophytes, bryophytes, lycophytes, ferns, gymnosperms and angiosperms, alongside FtsZ in cyanobacteria and FtsZA in red algae. We also provided functional evidences of the C-terminal motifs from CreZ1 (*Chlamydomonas reinhardtii* FtsZ1) and PpZ1B (*Physcomitrella patens* FtsZ1B) with AtFZ1 (*A. thaliana* FtsZ1) to regulate chloroplast division in vivo. Our results illuminated the functional differentiation between FtsZ1 and FtsZ2 during plant evolution.

## Results

### Evolutionary Characteristics of the FtsZ C-terminal Motif

**Fig. 1.**
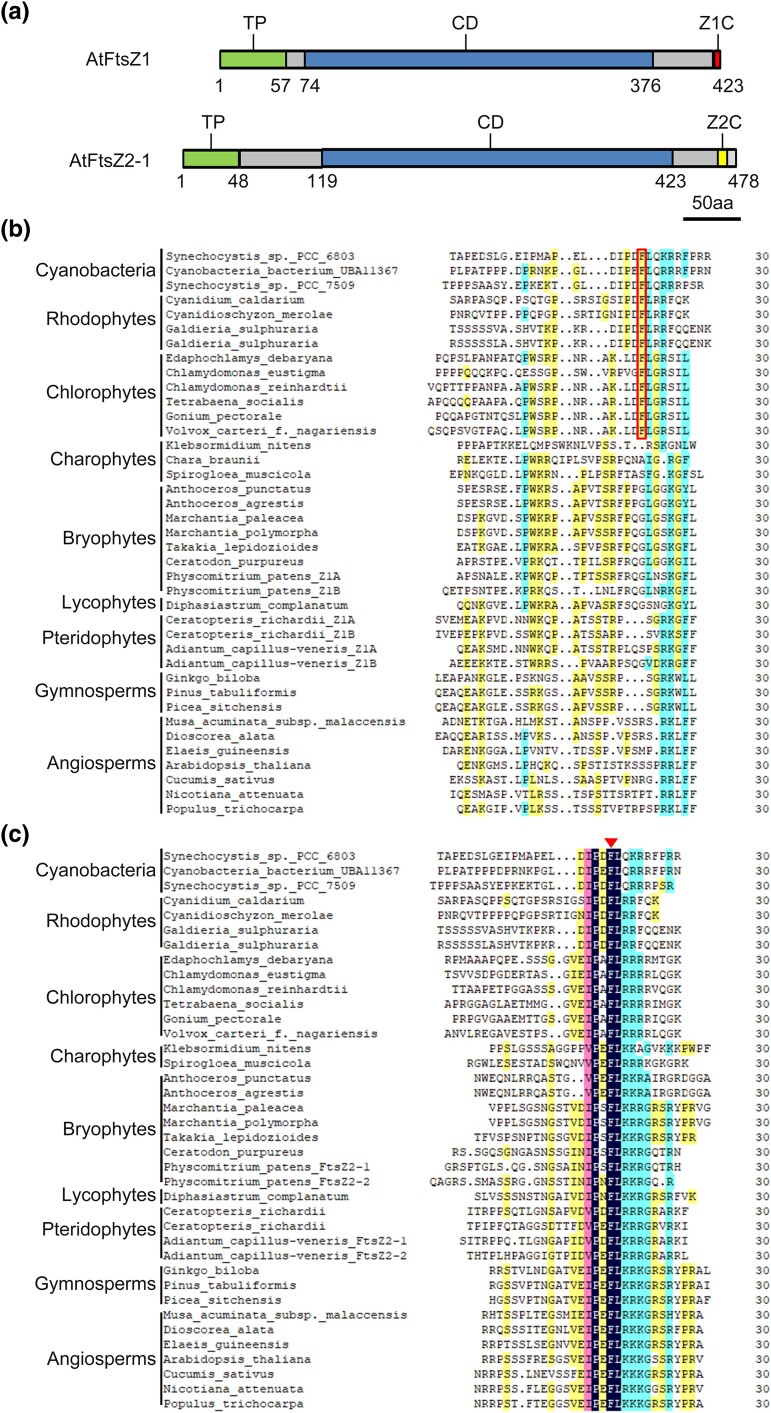
Different sequence motifs were evolved at the C-terminus of FtsZ1 and FtsZ2 during plant evolution. a) Diagrams of AtFtsZ1 and AtFtsZ2 protein domains. The predicted transit peptide (TP) is shown in green, the core domain (CD) is shown in blue, the C-terminal motif of AtFtsZ1 is shown (Z1C) in red, and the C-terminal motif of AtFtsZ2 (Z2C) is shown in yellow. (b and c) Multiple sequence alignments of the C-terminal 30 amino acids (C30aa) from Cyanobacterial FtsZ and Rhodophyta FtsZA, alongside FtsZ1 b) and FtsZ2 c) across various species, including Chlorophytes, Charophytes, Bryophytes, Lycophytes, Pteridophytes, Gymnosperms, and Angiosperms, arranged from top to bottom. Red box b) and red triangle c) indicate the key amino acid phenylalanine f) important for the interaction with ARC6 (Maple et al. 2005; Zhang et al. 2016).

FtsZ is conserved across cyanobacteria and plants, starting with a singular FtsZ in cyanobacteria and diverging into two distinct subfamilies: FtsZ1 and FtsZ2 in green algae and land plants, and FtsZB and FtsZA in red algae (supplementary fig. S1, Supplementary Material online). In *A. thaliana*, both AtFtsZ1 and AtFtsZ2-1 contain C-terminal motifs (Fig. 1a). However, their functions differ significantly (Zhang et al. 2016; Liu et al. 2022). To analyze the evolutionary trajectory of FtsZ, we aligned the C-terminal 30 amino acids (C30aa) of FtsZ in cyanobacteria and FtsZA in red algae with those of both FtsZ1 and FtsZ2 (Fig. 1b and c). The alignment revealed a core sequence, IPDFL, retained in FtsZ2 but shortened in FtsZ1 during the chlorophyta stage, eventually leading to a distinct motif in charophytes. This motif was simplified and became more conserved in angiosperms.

These findings indicate a divergence between FtsZ1 and FtsZ2, with the C-terminal motif of FtsZ1 evolving independently, resulting in distinct amino acid sequences at their C-termini and suggesting they play different roles in chloroplast division.

### The C-terminal Motif of FtsZ1 Exhibits Membrane-Binding Activity in Different Species

**Fig. 2.**
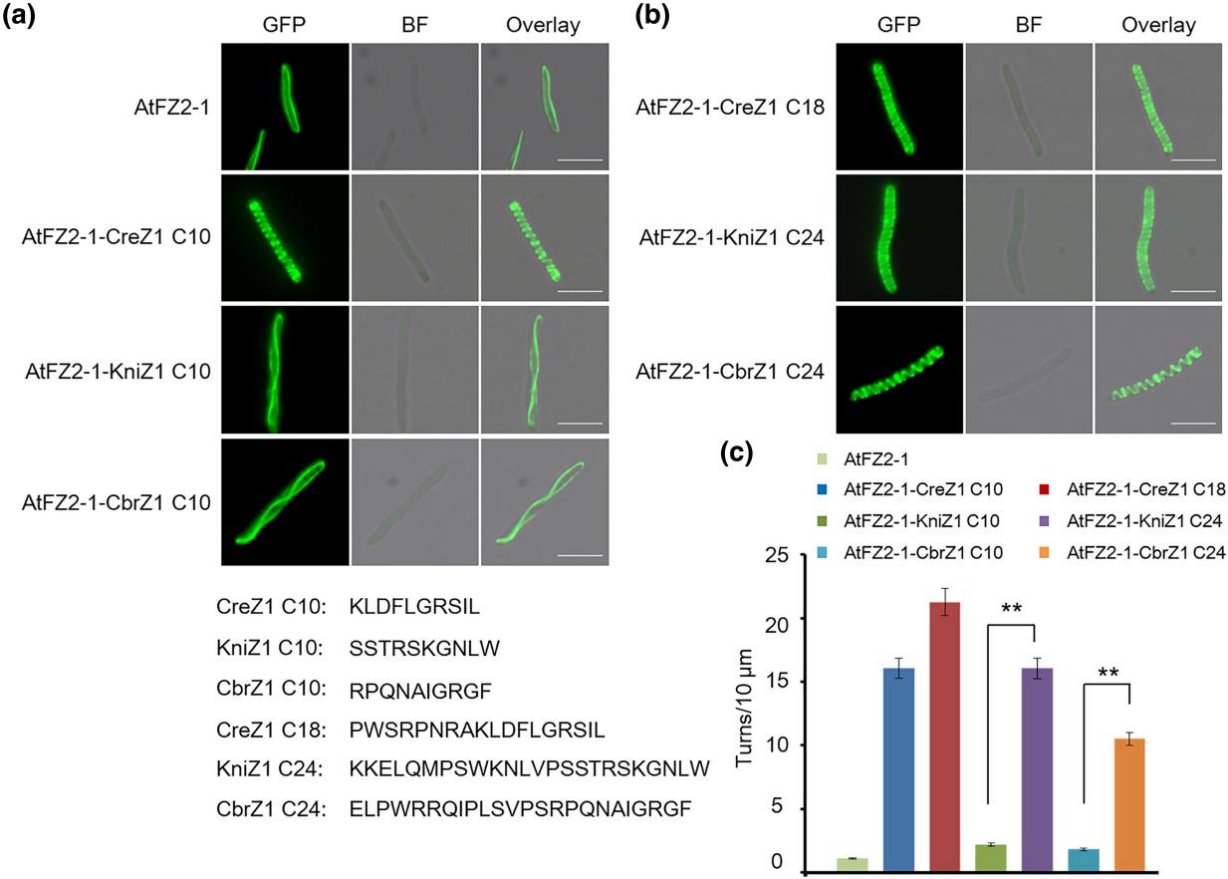
Green alga FtsZ1 C-terminal sequences have membrane-binding activity. a) GFP-AtFZ2-1 with a fusion of the C-terminal 10 amino acids of CreZ1, KniZ1 and CbrZ1 expressed in *E. coli*. Bars = 5 μm. b) GFP-AtFZ2-1 with fusion of longer sequences of the C-termini of CreZ1, KniZ1 and CbrZ1 formed helical structures in *E. coli*. Bars = 5 μm. c) Statistical analysis of the helical density of the FtsZ fusion proteins in a) and (b). *t* test, **P < 0.01. Error bars represent the mean ± SD.

Our previous research has shown that the C-terminal motif of AtFtsZ1 in *A. thaliana* exhibits membrane-binding activity, crucial for chloroplast division regulation (Liu et al. 2022). To examine whether this membrane-binding ability of the FtsZ1 C-terminal motif has persisted throughout evolution, we engineered fusion proteins by appending the C-terminal 10 amino acid residues from CreZ1 (*C. reinhardtii* FtsZ1), KniZ1 (*Klebsormidium nitens* FtsZ1), CbrZ1 (*Chara braunii* FtsZ1) and SmuZ1 (*Spirogloea muscicola* FtsZ1) to the GFP-tagged C-terminus of AtFtsZ2-1, and expressed these constructs in *E. coli* cells (Fig. 2a, and supplementary fig. S2, Supplementary Material online). The GFP-AtFtsZ2-1-CreZ1 C10 fusion formed dense, spiral-shaped structures with 16.07 spiral turns per 10 μm (Fig. 2a and c), indicating the membrane-binding activity of CreZ1 C10. Conversely, GFP-AtFtsZ2-1-KniZ1 C10, GFP-AtFtsZ2-1-CbrZ1 C10 and GFP-AtFtsZ2-1-SmuZ1 C10 produced spirals of much lower density with 2.19, 1.81 and 3.05 spiral turns per 10 μm, respectively (Fig. 2a and c, supplementary fig. S2a and b, Supplementary Material online). The result indicated that KniZ1 C10, CbrZ1 C10 and SmuZ1 C10 contain a low membrane-binding activity. Extending the C-terminal amino acids of CreZ1, KniZ1, CbrZ1 and SmuZ1 in the fusion with GFP-AtFtsZ2-1 resulted in significantly denser spiral structures for GFP-AtFtsZ2-1-CreZ1 C18, GFP-AtFtsZ2-1-KniZ1 C24, GFP-AtFtsZ2-1-CbrZ1 C24 and GFP-AtFtsZ2-1-SmuZ1 C24 with 21.26, 16.05, 10.5 and 6.1 spiral turns per 10 μm, respectively (Fig. 2b and c, and supplementary fig. S2a and b, Supplementary Material online). These findings suggest that the membrane-binding activity of FtsZ1’s C-terminal motifs in chlorophytes exists, but in charophytes, a longer amino acid sequence became necessary for this activity.

**Fig. 3.**
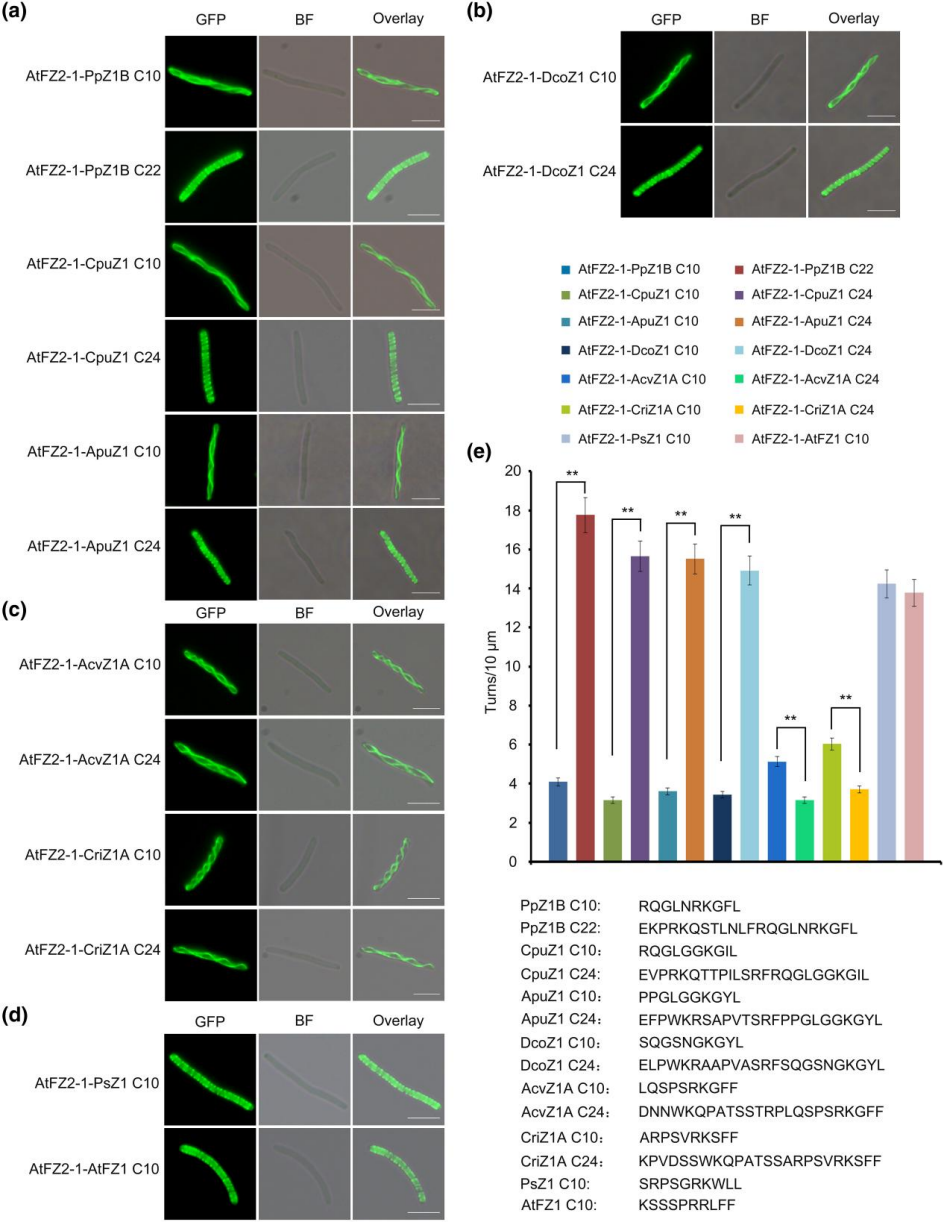
Land plant FtsZ1 C-terminal sequences have membrane-binding activity. C10, C22 and C24 represent the last 10, 22 and 24 amino acid residues of the protein, respectively. Shown at the lower right corner are the detailed amino acid sequences. a) Bryophyta FtsZ1 C-terminal sequences have membrane-binding activity. GFP-AtFZ2-1 with a fusion of PpZ1B C10, PpZ1B C22, CpuZ1 C10, CpuZ1 C24, ApuZ1 C10 and ApuZ1 C24 expressed in *E. coli* formed helical structures. Bars = 5 μm. b) Lycophyta FtsZ1 C-terminal sequences have membrane-binding activity. GFP-AtFZ2-1 with a fusion of DcoZ1 C10 and DcoZ1 C24 formed helical structures in *E. coli*. Bars = 5 μm. c) Fern FtsZ1 C-terminal sequences have membrane-binding activity. GFP-AtFZ2-1 with a fusion of AcvZ1A C10, CriZ1A C10, AcvZ1A C24 and CriZ1A C24 formed low helical structures in *E. coli*. Bars = 5 μm. d) Spermatophyta FtsZ1 C-terminal sequences have membrane-binding activity. GFP-AtFZ2-1 with a fusion of PsZ1 C10 and AtFZ1 C10 formed helical structures in *E. coli*. Bars = 5 μm. e) Statistical analysis of the helical density of the FtsZ fusion proteins in (A to D). *t* test, **P < 0.01. Error bars represent the mean ± SD.

**Fig. 4.**
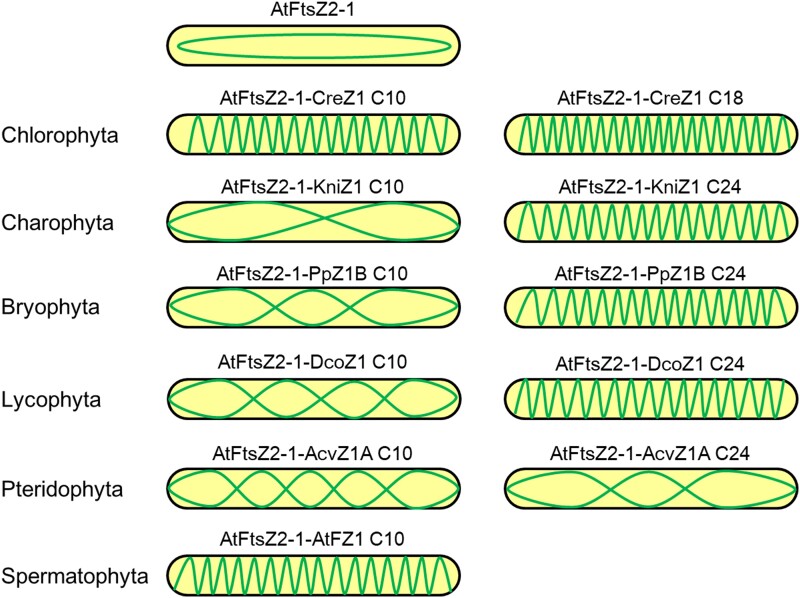
Pattern diagram of helical structures formed by FtsZ fusion proteins expressed in *E. coli*. The morphology of FtsZ2-1 is altered into spiral structures due to the membrane-binding activity of FtsZ1 C-terminal motifs in various species. The characteristic of FtsZ spiral density was significantly changed in Charophytes and Pteridophyta during plant evolution (Figs. 2 and 3).

We further investigate the C-terminal membrane-binding activity of FtsZ1 in a variety of other species, including hornwort (*Anthoceros punctatus* FtsZ1 [ApuZ1]), liverwort (*Marchantia paleacea* FtsZ1 [MpZ1]), mosses (*P. patens* FtsZ1B [PpZ1B] and *Ceratodon purpureus* FtsZ1 [CpuZ1]), lycophyte (*Diphasiastrum complanatum* FtsZ1 [DcoZ1]), ferns (*Adiantum capillus-veneris* FtsZ1A [AcvZ1A] and *Ceratopteris richardii* FtsZ1A [CriZ1A]), gymnosperm (*Picea sitchensis* FtsZ1 [PsZ1]), and angiosperm (*A. thaliana* FtsZ1 [AtFZ1]), by expressing their fusion proteins in *E. coli*. Our observations revealed that hornwort ApuZ1, liverwort MpZ1, moss PpZ1B and CpuZ1, and lycophyta DcoZ1 share spiral density characteristics with charophytes, requiring longer C-terminal amino acids for forming denser helical structures (Fig. 3a, b and e, and supplementary fig. S2a and b, Supplementary Material online). In contrast, the fusion proteins GFP-AtFZ2-1-PsZ1 C10 (14.23 spiral turns per 10 μm) and GFP-AtFZ2-1-AtFZ1 C10 (13.78 spiral turns per 10 μm) resembled those of GFP-AtFtsZ2-1-CreZ1 C10 (16.07 spiral turns per 10 μm), where shorter C-terminal motifs resulted in denser spirals (Fig. 3d and e). Interestingly, fusion proteins GFP-AtFZ2-1-AcvZ1A C10 (5.13 spiral turns per 10 μm) and GFP-AtFZ2-1-CriZ1A C10 (6.03 spiral turns per 10 μm) showed higher spiral densities compared with GFP-AtFZ2-1-AcvZ1A C24 (3.15 spiral turns per 10 μm) and GFP-AtFZ2-1-CriZ1A C24 (3.7 spiral turns per 10 μm), which significantly reduced helical densities and are very different from previous plants (Fig. 3c and e). During plant evolution, there is a trend for the C-terminal membrane-binding sequences of FtsZ1 to shorten and move to the C-terminal end in higher plant species (supplementary fig. S3, Supplementary Material online). These findings highlight the diverse membrane-binding capabilities and characteristics of the FtsZ1 C-terminal motifs across various species (Fig. 4).

**Fig. 5.**
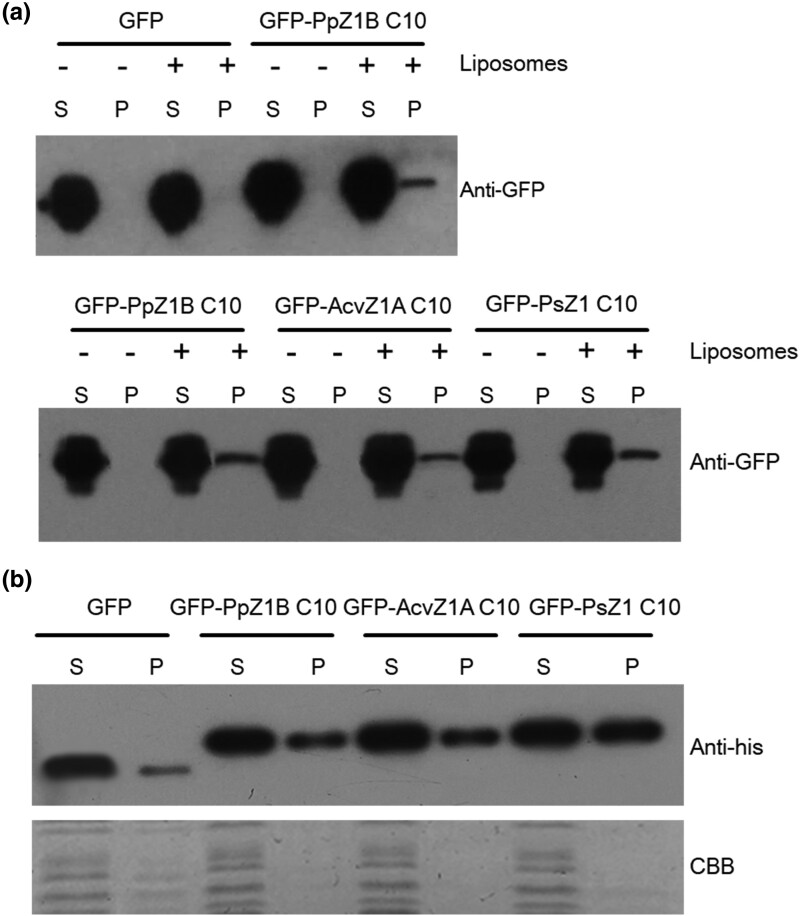
The C-terminal motifs of FtsZ1 in different species exhibit membrane-binding activity. a) Liposome co-precipitation with GFP, GFP-PpZ1B C10, GFP-AcvZ1A C10 and GFP-PsZ1 C10 in vitro. To analyze the membrane-binding activity of GFP, GFP-PpZ1B C10, GFP-AcvZ1A C10 and GFP-PsZ1 C10, the proteins were incubated with (+) liposomes or without (−) liposomes. Supernatant (S) and pellet (P) were separated by centrifugation. Immunoblots were analyzed by anti-GFP antibodies. b) Fractionation analysis of GFP fused with FtsZ C-termini from different plants. Proteins GFP, GFP-PpZ1B C10, GFP-AcvZ1A C10 and GFP-PsZ1 C10 were expressed in *E. coli*, and bacterial cells of equal quantity were lysed by lysozyme. Supernatant (S) and pellet (P) were separated by centrifugation. Immunoblots were analyzed by anti-his antibodies. Coomassie Brilliant Blue (CBB) staining was used as a loading control.

To further analyze the membrane-binding activity of the FtsZ1 C-terminal motifs from various species, we purified proteins GFP, GFP-PpZ1B C10, GFP-AcvZ1A C10 and GFP-PsZ1 C10 and then incubated them with liposomes. A part of the GFP-PpZ1B C10, GFP-AcvZ1A C10 and GFP-PsZ1 C10 was found to co-pellet with the liposomes, indicating a membrane-binding activity (Fig. 5a). Additionally, we investigated the relationship between membrane-binding efficacy and spiral density by expressing these proteins in bacteria, followed by lysis of equal amounts of bacteria with lysozyme. Detection of these proteins by anti-his antibodies revealed that a small fraction of GFP-PpZ1B C10 and GFP-AcvZ1A C10, and a larger fraction of GFP-PsZ1 C10, were associated with membrane (Fig. 5b).

These results indicate that the C-terminal motifs of FtsZ1 retain membrane-binding activity during the transition from algae to land plants. The unique membrane-binding characteristics observed in charophytes and ferns (Figs. 2 to 4) highlight their potential role as critical evolutionary junctions due to their distinct properties.

### RR/KLFF Motif is Important for the FtsZ1 Membrane Binding Activity in Various Species

**Fig. 6.**
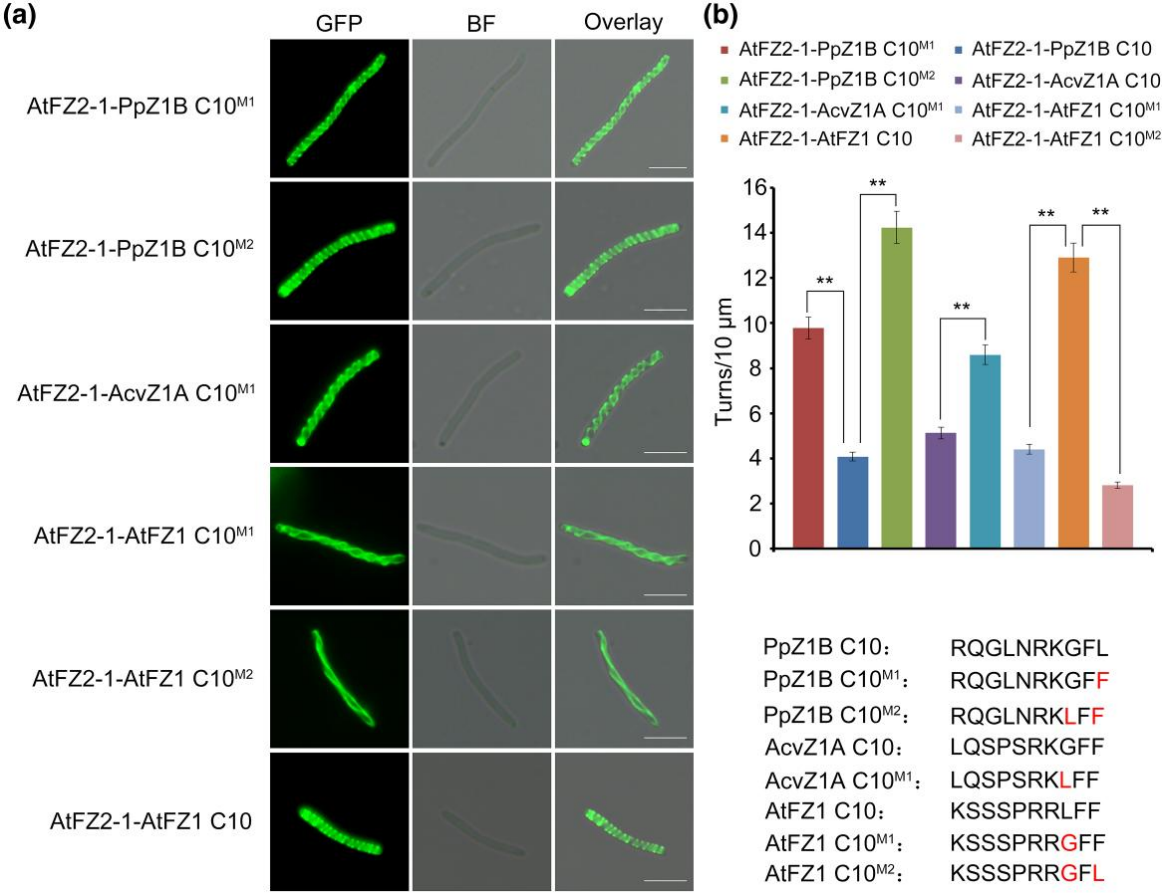
The RR/KLFF motif is important for the membrane binding activity of FtsZ1 in various angiosperm species. a) GFP-AtFZ2-1 with a fusion of PpZ1B C10^M1^, PpZ1B C10^M2^, AcvZ1A C10^M1^, AtFZ1 C10^M1^, AtFZ1 C10^M2^ and AtFZ1 C10 formed helical structure in *E. coli*. Scale bars = 5 μm. Shown at the lower right corner is the detailed amino acid sequences of PpZ1B C10^M1^, PpZ1B C10^M2^, AcvZ1A C10^M1^, AtFZ1 C10^M1^, AtFZ1 C10^M2^, and AtFZ1 C10. Mutations are highlighted in red. BF, Bright field. b) Statistical analysis of the helical density of the FtsZ fusion proteins in (a). *t* test, **P < 0.01. Error bars represent the mean ± SD.

To assess the role of the highly conserved C-terminal motif RR/KLFF in angiosperm FtsZ1 (Liu et al. 2022), for membrane-binding activity in other species, we modified the last five C-terminal amino acids of PpZ1B C10 from RKGFL to RKGFF, resulting in the mutant GFP-AtFZ2-1-PpZ1B C10^M1^. GFP-AtFZ2-1-PpZ1B C10^M1^ formed spiral structures in *E. coli* cells with 9.79 spiral turns per 10 μm, which notably increased in helical density compare to GFP-AtFZ2-1-PpZ1B C10 (4.08 spiral turns per 10 μm) (Fig. 6a and b). Similarly, we constructed mutant fusion proteins GFP-AtFZ2-1-PpZ1B C10^M2^ and GFP-AtFZ2-1-AcvZ1A C10^M1^ by changing their C-terminal five amino acids to RKLFF and expressed in *E. coli*. The fusion proteins GFP-AtFZ2-1-PpZ1B C10^M2^ and GFP-AtFZ2-1-AcvZ1A C10^M1^ formed helical structure with 14.23 and 8.6 spiral turns per 10 μm, respectively, showing significantly enhanced helical densities in bacteria (Fig. 6a and b).

Furthermore, we altered the last five C-terminal amino acids of AtFZ1 C10 from RRLFF to RRGFF (AtFZ1 C10^M1^) and RRGFL (AtFZ1 C10^M2^), inspired by the sequences found in AcvZ1A (RKGFF) and PpZ1B (RKGFL), respectively. These modifications resulted in a substantial decrease in helical density for both GFP-AtFZ2-1-AtFZ1 C10^M1^ and GFP-AtFZ2-1-AtFZ1 C10^M2^ in *E. coli* cells with 4.4 and 2.82 spiral turns per 10 μm, respectively (Fig. 6a and b).

These results not only indicate the significance of the RR/KLFF sequence in the C-terminal of angiosperm FtsZ1 for membrane binding activity across species, but also suggest an evolutionary direction for the FtsZ1 C-terminal motifs.

### The C-terminal Motif of FtsZ1 in Different Species has Similar Functions *in Vivo*

**Fig. 7.**
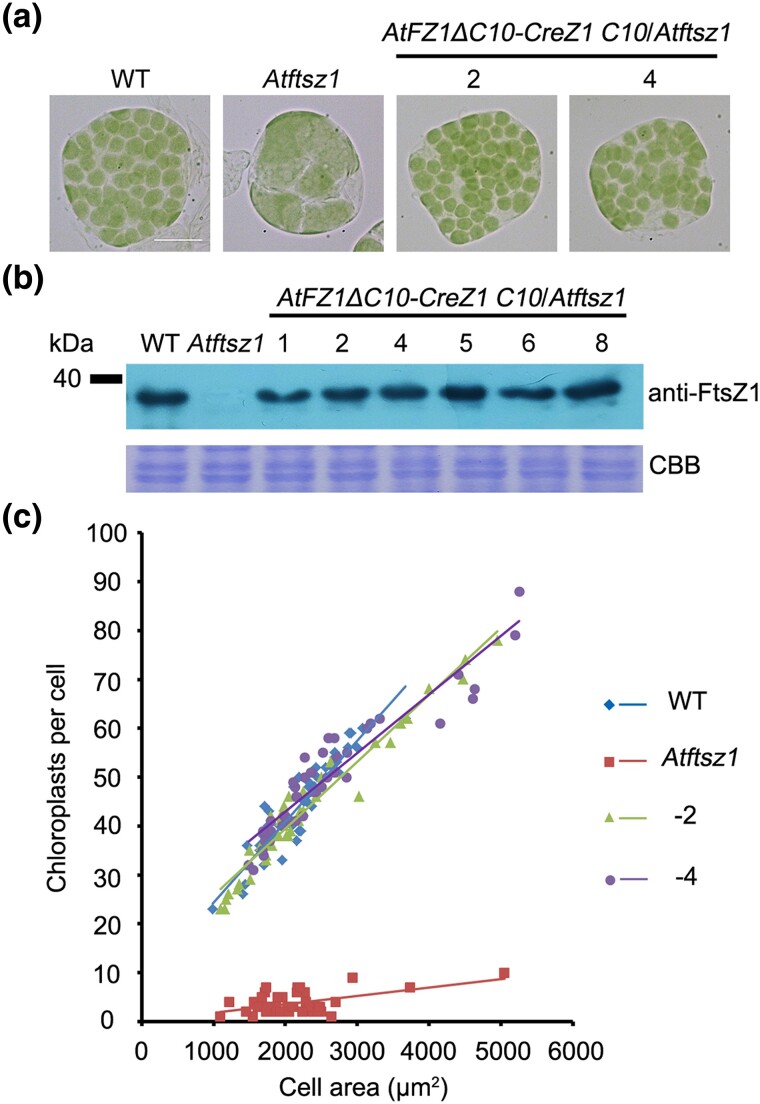
CreZ1 C10 has a function similar to AtFZ1 C10 in vivo. a) Chloroplast phenotype of wild type (WT), *Atftsz1* and transgenic plants. Scale bar = 10 μm. All the images have the same magnification. b) Immunoblot analysis of FtsZ1 protein levels in WT, *Atftsz1* and transgenic plants with anti-FtsZ1 antibodies. Coomassie Brilliant Blue (CBB) staining served as a loading control. c) Correlation between chloroplast number and cell area in WT, *Atftsz1*, and transgenic plants shown in a). The best-fit lines had slopes of 0.0166 (*R*^2^ = 0.8633), 0.0017 (*R*^2^ = 0.2697), 0.0137 (*R*^2^ = 0.9486), and 0.012 (*R*^2^ = 0.879) for the wild type, *Atftsz1*, and two transgenic lines, respectively. *n* > 30 cells for each sample.

To assess the in vivo functionalities of FtsZ1's C-terminal motifs from various species, we engineered a fusion protein by replacing the last 10 amino acids of AtFZ1 (AtFZ1 C10) with that of CreZ1 (CreZ1 C10). *AtFZ1ΔC10-CreZ1 C10* was introduced into an *Atftsz1* null mutant under the control of the native *AtFtsZ1* promoter. Remarkably, the chloroplast phenotype of the *Atftsz1* mutant was rescued by *AtFZ1ΔC10-CreZ1 C10* (Fig. 7a). Western blot analysis confirmed that the FtsZ1 protein levels in the transgenic plants were comparable to those in the wild type, and the number of chloroplasts per cell is similar to that of the wild type (Fig. 7b and c).

**Fig. 8.**
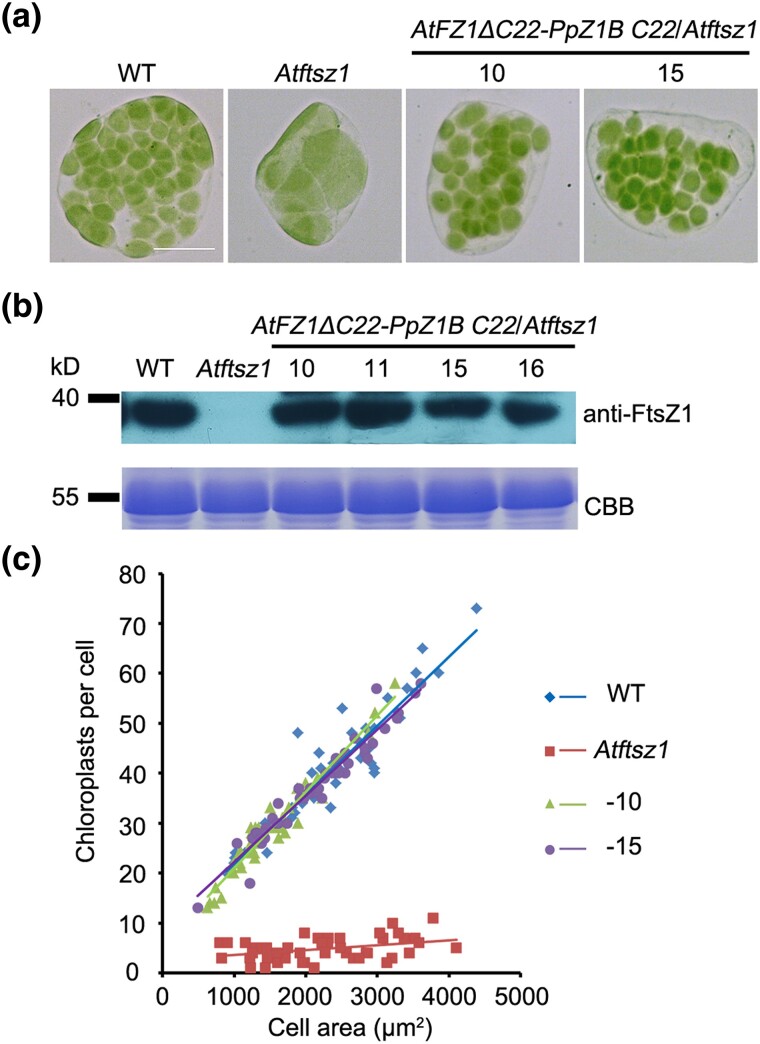
PpZ1B C22 has a function similar to AtFZ1 C22 in vivo. a) Chloroplast phenotype in wild type (WT), *Atftsz1* and transgenic plants. Scale bar = 10 μm. All the images have the same magnification. b) Immunoblot analysis of FtsZ1 level in WT, *Atftsz1* and transgenic plants with anti-FtsZ1 antibodies. Coomassie Brilliant Blue (CBB) staining served as a loading control. c) Correlation between chloroplast number and cell area in WT, *Atftsz1*, and transgenic plants shown in a). The best-fit lines had slopes of 0.0139 (*R*^2^ = 0.8836), 0.001 (*R*^2^ = 0.1408), 0.0153 (*R*^2^ = 0.9399), and 0.0133 (*R*^2^ = 0.9423) for the wild type, *Atftsz1*, and two transgenic plant lines, respectively. *n* > 30 cells for each sample.

Similarly, we constructed *AtFZ1ΔC22-PpZ1B C22*, a fusion protein in which AtFZ1's last 22 amino acids (AtFZ1 C22) were replaced with that of PpZ1B (PpZ1B C22), and introduced this construct into the *Atftsz1* mutant with the native promoter of *AtFtsZ1*. The chloroplast phenotype in plants transformed with *AtFZ1ΔC22-PpZ1B C22* was effectively rescued (Fig. 8a). Further analyzes, including immunoblotting with anti-FtsZ1 antibodies, showed that many transgenic lines had FtsZ1 protein levels and chloroplast numbers per cell close to those of the wild type (Fig. 8b and c).

Immunofluorescence staining with anti-FtsZ2-1 antibodies revealed that, unlike in the *Atftsz1* mutant where long FtsZ filaments and multiple rings were observed, FtsZ in transgenic plants typically formed a single ring at the division site, similar to those observed in the wild-type (supplementary fig. S4, Supplementary Material online).

Despite the varying amino acid sequences, the C-terminal motifs of FtsZ1 from different species have similar functions in chloroplast division regulation throughout plant evolution.

### FtsZ C-terminal Motifs in Cyanobacteria and Algae Exhibit Both Protein Interaction and Membrane Binding Activity

FtsZ1 and FtsZ2 were derived from cyanobacteria FtsZ (Stokes and Osteryoung 2003). Previous studies have reported that the C-terminus of SynFZ (*Synechocystis sp. PCC6803* FtsZ), CmeFZA (*Cyanidioschyzon merolae* FtsZA) and AtFtsZ2 interact with the membrane protein Ftn2 in cyanobacteria or its homolog ARC6 in plants (Zhang et al. 2016; Yoshida 2018; Camargo et al. 2019). To investigate whether the C-terminal motifs of SynFtsZ and CmeFZA have membrane-binding activity, fusion proteins GFP-AtFZ2-1-SynFZ C10 and GFP-AtFZ2-1-CmeFZA C10 were expressed in *E. coli* cells and the fusion proteins were found to form dense helical structures, suggesting they have membrane-binding activity (supplementary fig. S5, Supplementary Material online). Thus, SynFZ C10 and CmeFZA C10 have both membrane-binding activity and protein interaction function.

**Fig. 9.**
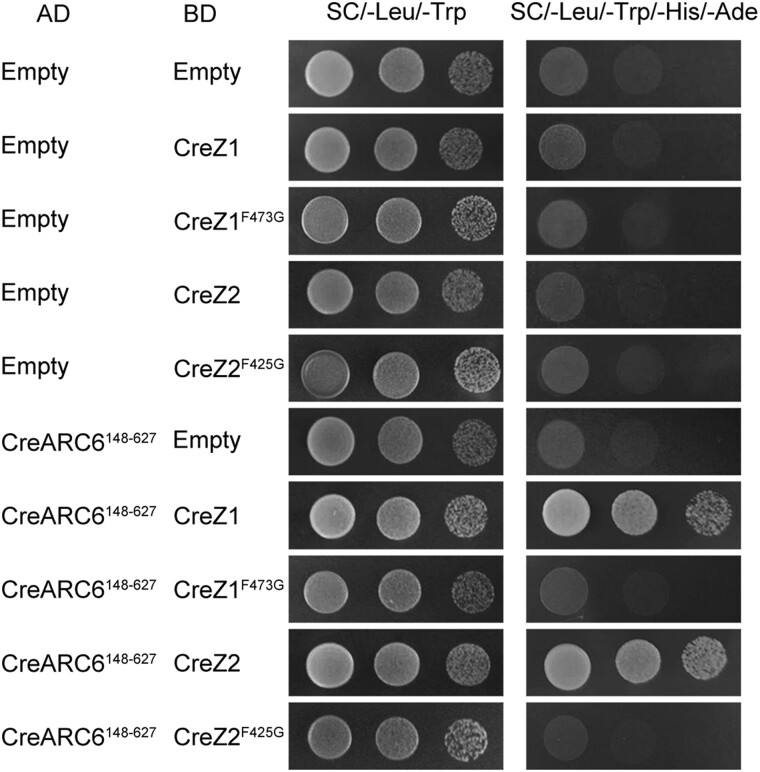
CreZ1 and CreZ2 can interact with CreARC6. To analyze the interaction relationship of both CreZ1 and CreZ2 with CreARC6^148–627^ by yeast two-hybrid system, the encoding sequence of CreARC6^148–627^ was cloned into pGADT7 (AD) vector, encoding sequences of CreZ1 and CreZ2 were cloned into pGBKT7 (BD) vectors. CreZ1^F473G^, phenylalanine^473^ (F) mutated to glycine (G). CreZ2^F425G^, phenylalanine^425^ (F) mutated to glycine (G). The key residues of CreZ1 and CreZ2 for the mutational analysis are also shown in Fig. 1 b) and c), respectively. Yeast was cultured on synthetic dropout medium (−Leu/–Trp) and selective medium (Leu/–Trp/–His/-Ade) across three gradient dilutions 1, 10^−1^ and 10^−2^.

FtsZ diverged into FtsZ1 and FtsZ2 during the chlorophyta stage. To explore the presence of membrane-binding activity and protein interaction function in these proteins from green algae, we constructed a fusion protein, GFP-CreZ2 (*C. reinhardtii* FtsZ2), and expressed it in *E. coli* cells. Despite the significant agglomeration, the fusion protein formed a spiral structure, suggesting that CreZ2 still exhibits membrane-binding activity (supplementary fig. S5, Supplementary Material online). Further, we assessed the interactions between CreZ1, CreZ2, and CreARC6 (*C. reinhardtii* ARC6) using a yeast two-hybrid assay. The result revealed that both CreZ1 and CreZ2 interact with CreARC6 (Fig. 9). The essential amino acid phenylalanine (F), crucial for the interaction between FtsZ2 and ARC6 (Maple et al. 2005; Zhang et al. 2016), exists in the C-terminal motifs of both CreZ1 and CreZ2 (Fig. 1b and c). Mutating phenylalanine (F) to glycine (G) for CreZ1^F473G^ and CreZ2^F425G^ resulted in loss of interaction with CreARC6 (Fig. 9).

These findings demonstrate that the C-terminal motifs of SynFZ and CmeFZA exhibit both membrane-binding activity and protein-protein interaction functions. At the initial stage of FtsZ differentiation in chlorophytes, FtsZ1 and FtsZ2 continue to retain these crucial functions.

### Higher Plant FtsZ1 C-terminal Motif Lost Protein Interaction Ability

**Fig. 10.**
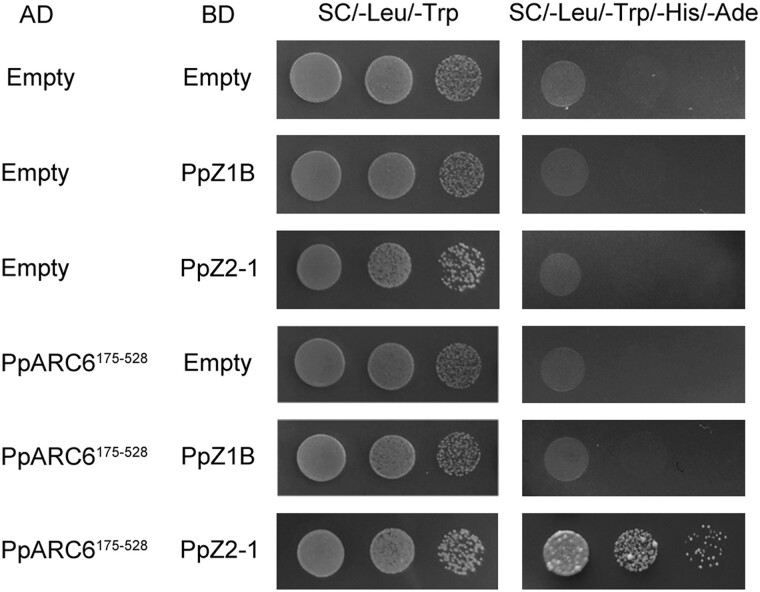
PpZ1B does not interact with PpARC6. To analyze the interaction relationship of both PpZ1B and PpZ2-1 with PpARC6^175–528^ by yeast two-hybrid analysis, the encoding sequence of PpARC6^175–528^ was cloned into pGADT7 (AD) vector, encoding sequences of PpZ1B and PpZ2-1 were cloned into pGBKT7 (BD) vectors. Yeast was cultured on synthetic dropout medium (−Leu/–Trp) and selective medium (Leu/–Trp/–His/-Ade) across three gradient dilutions 1, 10^−1^ and 10^−2^.

The crucial amino acid Phenylalanine (F), essential for the interaction of FtsZ2 with the membrane protein ARC6, is absent in the C-terminus of FtsZ1 in charophytes and higher plants, as shown in sequence alignments (Fig. 1b and supplementary fig. S3, Supplementary Material online). In contrast, FtsZ2 retains the Phenylalanine throughout evolution process (Fig. 1c). To investigate whether FtsZ1 from higher plants lacks interaction with ARC6, we conducted a yeast two-hybrid assay, and the results revealed that PpZ1B does not interact with PpARC6 (*P. patens* ARC6), whereas PpZ2-1 (*P. patens* FtsZ2-1) does interact with PpARC6 (Fig. 10).

To further investigate the membrane-binding activity of FtsZ2 during plant evolution, we expressed the fusion protein GFP-PpZ2-1 in bacteria and observed a high-density spiral structure (supplementary fig. S5, Supplementary Material online), indicating that PpZ2-1 contains membrane-binding activity. We also expressed GFP-AcvZ2-1 (*Adiantum capillus-veneris* FtsZ2-1) in *E. coli* cells, which formed straight filaments (supplementary fig. S5, Supplementary Material online), suggesting a loss of membrane-binding activity in AcvZ2-1.

## Discussion

**Fig. 11.**
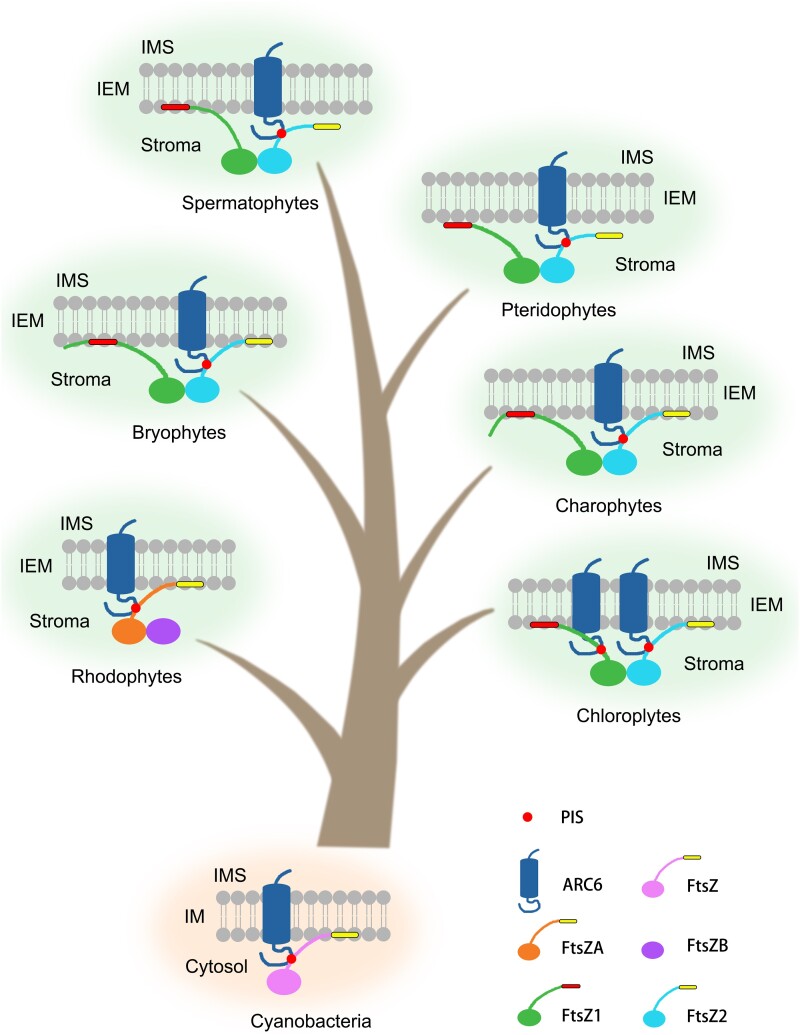
A schematic diagram of the evolution and functional differentiation of FtsZ C-terminal motifs during plant evolution. In cyanobacteria, the C-terminal motif of FtsZ interacts with Ftn2 (homolog of ARC6) and binds with cyanobacterial inner envelope membrane. In red algae, the C-terminal motif of FtsZA interacts with ARC6 and binds with chloroplast inner envelope membrane, FtsZB lacks C-terminal structure. In chlorophytes, the C-terminal motifs of FtsZ1 and FtsZ2 interact with ARC6 and bind with chloroplast inner envelope membrane. In charophytes and higher plants, FtsZ1 lost the ability to interact with ARC6. In vascular plants, FtsZ2 lost membrane-binding activity. The red dots denote the key protein interaction site (PIS) phenylalanine (F) within the FtsZ C-terminal motif, crucial for the interaction with the membrane protein ARC6. It is noteworthy that during evolution, the membrane-binding motifs of FtsZ1 become shorter and closer to the C-terminal end. IEM, inner envelope membrane; IMS, inter membrane space; IM, inner membrane.

In this study, our results revealed an evolutionary route that delineates the functional characters of the FtsZ C-terminal motifs throughout plant evolution (Fig. 11). In chloroplytes, both FtsZ1 and FtsZ2 retain the ability to interact with ARC6. As evolution progressed, FtsZ1 in species beyond the chloroplyta stage lost this interaction capability with ARC6. FtsZ2 maintained the interaction capability with ARC6 up to seed plants, indicating a preservation of this function throughout the plant evolution (Miyagishima 2011). For membrane-binding activity, FtsZ1 exhibits this capability up to seed plants. In contrast, FtsZ2's membrane-binding activity is retained until bryophytes, after which it is lost, marking a specific evolutionary shift in the functional repertoire of the FtsZ proteins.

During evolution, FtsZ1's C-terminal motifs show flexibility and diversity, adapting readily, while FtsZ2's C-terminal motifs remain stable (Fig. 1b and c). These evolutionary distinctions are closely linked to their functional roles (Zhang et al. 2016; Liu et al. 2022). We have verified the membrane-binding activity of FtsZ1 C-terminal motif of various species in *E. coli* and in vitro experiments (Figs. 2 to 5, and supplementary fig. S2, Supplementary Material online), and revealing significant variability in their membrane-binding sequences across species. FtsZ1 exhibits dynamic functionality, playing a pivotal role in exerting the contraction force necessary for chloroplast division (TerBush and Osteryoung 2012; TerBush et al. 2018). The C-terminal motif of FtsZ1 is essential for its membrane binding ability and requires shorter sequence at the C-terminal end to effectively interact with the chloroplast membrane (supplementary fig. S3, Supplementary Material online). The evolutionary pattern of the Z1C motif in plants is likely more favorable due to the fewer amino acids needed for effective membrane binding while still allowing for adequate turnover and maintaining its function in generating contraction forces for chloroplast division (TerBush and Osteryoung 2012; Liu et al. 2022).

Our findings indicate that the functions of membrane binding and protein interaction observed in FtsZ1 and FtsZ2 are inherited from cyanobacterial FtsZ C-terminal motifs (supplementary fig. S5, Supplementary Material online) (Mazouni et al. 2004). Initially, in chloroplytes, FtsZ differentiated into FtsZ1 and FtsZ2, retaining both membrane-binding and protein interaction functions (Figs. 2 and 9 and supplementary fig. S5, Supplementary Material online). Subsequently, the C-termini of FtsZ1 and FtsZ2 underwent functional differentiation. This divergence likely reflects evolution of specialized chloroplast division proteins like PLASTID DIVISION2 (PDV2) in streptophytes, MULTIPLE CHLOROPLAST DIVISION SITE1 (MCD1) in bryophytes, and PLASTID DIVISION1 (PDV1) and PARALOG OF ARC6 (PARC6) in ferns, whose impacts on FtsZ functionality requires further exploration (Miyagishima 2011; Miyagishima et al. 2011; Liu et al. 2024).

The envelope of chloroplasts changes during the evolution of plants. Cyanobacteria have a well-defined cell wall which is mostly made up of peptidoglycan (Leganés et al. 2005). The cell wall is absent in the chloroplast of red algae, green algae and other plants (Takano and Takechi 2010; Hirano et al. 2016). However, the peptidoglycan biosynthesis pathway was found to be well-conserved in bryophytes and lycophytes (Izumi et al. 2003; Dowson et al. 2022). Mutations of *Mur* (*murein*) genes in this pathway in *P. patens* affected chloroplast division (Machida et al. 2006). The application of penicillin, which can inhibit the biosynthesis of peptidoglycan, also blocked chloroplast division in bryophytes and lycophytes (Izumi et al. 2003; Machida et al. 2006; Takahashi et al. 2016). The *MurE* gene, although exists in seed plants, is not involved in chloroplast division any more (Garcia et al. 2008; Lin et al. 2017). Moreover, penicillin doesn’t inhibit the division of chloroplasts in seed plants either (Kasten and Reski 1997). In this work, the C-terminus of FtsZ1 in charophytes, bryophytes, and lycophytes required longer amino acid sequences to provide membrane-binding activity (Figs. 2 and 3 and supplementary fig. S2, Supplementary Material online). While the C-terminal membrane binding activity of FtsZ1 in seed plants was provided by a shorter motif at the very end of the C-terminus (Fig. 3 and supplementary fig. S3, Supplementary Material online). Unfortunately, the related study is lacking in ferns. The significant change of the membrane-binding sequences at the FtsZ1 C-termini from bryophytes and lycophytes to seed plants could be an adaption to the change of the peptidoglycan biosynthesis-dependent chloroplast division mechanism.

This study significantly advances the understanding of the functional differentiation of FtsZ C-terminal motifs across various species during evolution. It reveals the distinctive evolutionary paths and functional divergences of FtsZ1 and FtsZ2 C-terminal motifs throughout plant evolution (Fig. 11). The study traces the origin of the membrane-binding activity of the FtsZ1 C-terminal motif and the protein interaction function of the FtsZ2 C-terminal motif to the ancestral cyanobacterial FtsZ C-terminal motif. Initially, in chlorophytes, FtsZ differentiated into FtsZ1 and FtsZ2, with both variants maintaining dual functional capabilities (Figs. 2 and 9). The evolutionary forces behind the loss of the protein interaction function in streptophyta FtsZ1 and the loss of the membrane-binding activity in fern FtsZ2 are not fully understood. Further investigations in the future are essential to explore the evolution of these characters across species, offering insights into the adaptive mechanisms underlying chloroplast division.

## Materials and Methods

### Plant Materials

*Arabidopsis thaliana* ecotype Col-0, *C. reinhardtii* strain cc124, *P. patens* strain Gransden 2004 and *Adiantum capillus-veneris* were used in this study. *C. reinhardtii* strain cc124 (Harris 1989) and *P. patens* strain Gransden 2004 (Strepp et al. 1998) were cultivated as described previously. The temperature of the plant cultivation environment was 22 °C, the relative humidity was 40% to 60%, and the light-dark cycle was 16-h light/8-h dark.

### Bioinformatic Analysis

FtsZ protein sequences (cyanobacterial FtsZ, red alga FtsZA and FtsZB, FtsZ1 and FtsZ2 in various other species) were identified and downloaded from National Center for Biotechnology Information (https://blast.ncbi.nlm.nih.gov/Blast.cgi). Amino acid sequences of FtsZ C-terminal motifs analyzed in this study are shown in supplementary table S1, Supplementary Material online. The accession numbers of FtsZs are shown in supplementary table S2, Supplementary Material online. All the FtsZ protein sequences are shown in supplementary data, Supplementary Material online. Multiple sequence alignment of FtsZs C-terminal 30 amino acids in various species was performed using BioEdit software (version 7.2.5) (https://bioedit.software.informer.com/). Phylogenetic analysis of FtsZ proteins was performed using the maximum likelihood method of MEGA7 (https://megasoftware.net/).

### RNA-Isolation and RT-PCR

RNA-isolation of *A. thaliana*, *C. reinhardtii*, *P. patens* and *A. capillus-veneris* was performed using Trizol reagent (Aidlab Biotechnoligies, Beijing). The cDNA was synthesized using M5 First Strand cDNA Synthesis Kit (Mei5 Biotechnology, Beijing) with 2 μg total RNA.

### Plasmid Constructions

To analyze the membrane-binding activity of the Z1C motifs of various species, various plasmids were constructed for protein expression in *E. coli* BL21 (DE3). To construct *GFP-AtFZ2-1*, *GFP-CreZ2*, *GFP-PpZ2-1* and *GFP-AcvZ2-1*, cDNAs of *A. thaliana*, *C. reinhardtii*, *P. patens* and *A. capillus-veneris* were used as templates for the PCR-amplification using the primers AtFZ2-1-43 and AtFZ2-1-44, CreZ2-1 and CreZ2-2, PpZ2-1-4 and PpZ2-1-5, AcvZ2-1-3 and AcvZ2-1-5, respectively. These PCR products were digested with EcoRⅠ and XhoⅠ, except PpZ2-1, which was digested with KpnⅠ and XhoⅠ, and then ligated with GFP and cloned into pET-28a vector. To construct *GFP-AtFZ2-1-CreZ1 C10*, *GFP-AtFZ2-1-KniZ1 C10*, *GFP-AtFZ2-1-CbrZ1 C10*, *GFP-AtFZ2-1-SmuZ1 C10*, *GFP-AtFZ2-1-SynFZ C10*, *GFP-AtFZ2-1-CmeFZA C10*, *GFP-AtFZ2-1-ApuZ1 C10*, *GFP-AtFZ2-1-MpZ1 C10*, *GFP-AtFZ2-1-CpuZ1 C10*, *GFP-AtFZ2-1-PpZ1B C10*, *GFP-AtFZ2-1-DcoZ1 C10*, *GFP-AtFZ2-1-AcvZ1A C10*, *GFP-AtFZ2-1-CriZ1A C10*, *GFP-AtFZ2-1-PsZ1 C10* and *GFP-AtFZ2-1-AtFZ1 C10*, *GFP-AtFZ2-1* were PCR-amplified using the forward primer NcoⅠGFP, and reverse primers CreZ1-1, KniZ1-1, CbrZ1-1, SmuZ1-1, SynFZ-1, CmeFZA-1, ApuZ1-1, MpZ1-1, CpuZ1-3, PpZ1B-1, DcoZ1-1, AcvZ1A-1, CriZ1A-1, PsZ1-1, AtFZ1-46. These PCR products were digested with NcoⅠ and XhoⅠ and cloned into pET-28a vector. To construct *GFP-AtFZ2-1-PpZ1B C10^M1^*, *GFP-AtFZ2-1-PpZ1B C10^M2^*, *GFP-AtFZ2-1-AcvZ1A C10^M1^*, *GFP-AtFZ2-1-AtFZ1 C10^M1^*, *GFP-AtFZ2-1-AtFZ1 C10^M2^*, *GFP-AtFZ2-1-PpZ1B*, *GFP-AtFZ2-1-AcvZ1A* and *GFP-AtFZ2-1-AtFZ1* were PCR-amplified using the forward primer NcoⅠGFP, and reverse primers PpZ1B-M1, PpZ1B-M2, AcvZ1A-M1, AtFZ1C-M1 and AtFZ1C-M2, respectively. These PCR products were digested with NcoⅠ and XhoⅠ and cloned into pET-28a vector. To construct *GFP-AtFZ2-1-CreZ1 C18*, *GFP-AtFZ2-1-KniZ1 C24*, *GFP-AtFZ2-1-CbrZ1 C24*, *GFP-AtFZ2-1-SmuZ1 C24*, *GFP-AtFZ2-1-ApuZ1 C24*, *GFP-AtFZ2-1-MpZ1 C24*, *GFP-AtFZ2-1-PpZ1B C22*, *GFP-AtFZ2-1-CpuZ1 C24*, *GFP-AtFZ2-1-DcoZ1 C24*, *GFP-AtFZ2-1-CriZ1A C24*, *GFP-AtFZ2-1-AcvZ1A C24*, *GFP-AtFZ2-1* were PCR-amplified using the forward primer NcoⅠGFP, and reverse primers CreZ1-2, KniZ1-2, CbrZ1-2, SmuZ1-2, ApuZ1-2, MpZ1-2, PpZ1B-2, CpuZ1-1, DcoZ1-2, CriZ1A-2, AcvZ1A-2, respectively, and then the above PCR products were further amplified with the forward primer NcoⅠGFP, and reverse primers CreZ1-3, KniZ1-3, CbrZ1-3, SmuZ1-3, ApuZ1-3, MpZ1-3, PpZ1B-3, CpuZ1-2, DcoZ1-3, CriZ1A-3, AcvZ1A-3, respectively. The primer sequences are shown in supplementary table S3, Supplementary Material online. These PCR products were digested with NcoⅠ and XhoⅠ. All the digested products were ligated into pET-28a between the NcoI and XhoⅠ sites. *E. coli* cells with the above plasmids were cultured in LB medium with 50 mg·L^−1^ kanamycin, induced with 0.5 mM Isopropyl-β-D-1-thiogalactopyranoside (IPTG) for 8 h at 20 °C for fluorescence microscopy observation. The amino acid sequences of FtsZ C-terminal motifs are shown in supplementary table S1, Supplementary Material online.

To obtain complementation constructs *PFtsZ1: AtFtsZ1ΔC10-CreZ1 C10* and *PFtsZ1: AtFtsZ1ΔC10-PpZ1B C22*, genomic DNA of *A. thaliana* was amplified with forward primer AtFZ1-10 and reverse primers CreZ1-11 and PpZ1B-4, and then the above PCR products were further amplified with forward primer AtFZ1-10 and reverse primers CreZ1-12 and PpZ1B-5, respectively. The PCR products were digested with BamHⅠ and NcoⅠ, and then cloned into 3302Y2 vector. These constructs were transformed into an *AtFtsZ1* null mutant (Liu et al. 2022) by floral dipping method. The primer sequences are shown in supplementary table S3, Supplementary Material online.

### Fluorescence Microscopy and Image Analysis

The fusion proteins expressed in *E. coli* BL21 (DE3) cells were observed with a fluorescence microscope (NE910, Nexcope, Ningbo, China) equipped with a camera (E3ISPM). Bacterial cells were observed with an oil immersion 100× objective. Immunofluorescence staining was performed as described previously (Li et al. 2016) with the FtsZ2-1 antibodies (Liu et al. 2022). Images were analyzed with ImageJ (http://rsbweb.nih.gov/ij/; version 1.52v) and Photoshop (Adobe Photoshop CC 2015) softwares.

### Protein Expression and Purification

To express GFP, GFP-PpZ1B C10, GFP-AvcZ1A C10, GFP-PsZ1 C10 proteins with 6×His tag, PCR-amplifications were performed with the primers GFPEcoRⅠ and AtFZ2-1-46, and plasmids *pET-28a-GFP-AtFZ2-1*, *pET-28a-GFP-AtFZ2-1-PpZ1B C10*, *pET-28a-GFP-AtFZ2-1-AcvZ1A C10* and *pET-28a-GFP-AtFZ2-1-PsZ1 C10* as the templates, respectively. The primers sequences are shown in supplementary table S3, Supplementary Material online. The PCR products were digested with EcoRⅠ and ligated with T4 ligases (NEB), and then amplified with primers NcoⅠGFP and T7ter, the PCR products were digested with NcoⅠ and XhoⅠ and then cloned into pET30a expression vectors. The fusion proteins were expressed in *E. coli* BL21 strains with a His tag. Protein purification was performed using the same protocol as described previously (Liu et al. 2022).

### Liposome Co-Sedimentation and Membrane-binding Ability Analysis

Liposome preparation was performed as described previously (Liu et al. 2022). Proteins GFP, GFP-PpZ1B C10, GFP-AvcZ1A C10, GFP-PsZ1 C10 were incubated with liposomes or PBS (135 mM NaCl, 2.7 mM KCl, 10 mM Na_2_HPO_4_, 2 mM KH_2_PO_4_, pH 7.4) buffer for 1 h at room temperature. The mixtures were separated into supernatant and pellet with a centrifugation at 15,000 g for 10 min at room temperature, and then the pellet was washed with PBS for one time. The supernatant and pellet were probed by immunoblot with anti-GFP antibodies (Biodragon Beijing).

To analyze the relationship between membrane-binding ability and spiral density, proteins GFP, GFP-PpZ1B C10, GFP-AvcZ1A C10, GFP-PsZ1 C10 were expressed in *E. coli* BL21 strains in 20 mL LB medium with 50 mg·L^−1^ kanamycin, grown to an OD_600_ value of ∼0.45 at 37 °C, and then induced with 0.1 mM Isopropyl-β-D-1-thiogalactopyranoside (IPTG) for 2 h at 20 °C to OD_600_ value of ∼0.65. Bacterial cells of equal quantity were collected by centrifugation at 12,000 g for 1 min at room temperature, and resuspended with 500 μL TBS (20 mM Tris, 150 mM NaCl, pH 7.5), and then lysed for 2 min at room temperature after the addition of 50 μL lysozyme (ACE Biotechnology). Supernatants and pellets were separated with a centrifugation at 12,000 g for 5 min at room temperature, and then pellets were washed with TBS for one time. The supernatants and pellets were probed by immunoblot with anti-his antibodies (Jiaxuan biotech).

### Chloroplast Phenotype Analysis

To observe the chloroplast phenotype, *Arabidopsis* leaves from 4-week-old plants were fixed with 3.5% glutaraldehyde in darkness for 1 h at room temperature. Then, the glutaraldehyde was replaced with 0.1 M Na_2_EDTA (pH = 9.0) and the samples were incubated in a 55 °C water bath for 2 h. The images were captured with an Olympus CX21 (Olympus, Tokyo) microscope coupled with a USB 2.0 digital camera (Changheng, Beijing). The number of chloroplasts per cell was counted and analyzed with Excel (Microsoft).

### Immunoblot Analysis of Proteins in Transgenic Plants

Proteins were extracted from leaves of 4-week-old plants, and separated by SDS-PAGE gels. The total proteins were transferred to PVDF membrane (Bio-Rad), and then blocked with 5% (w/v) fat-free milk in TBST buffer (10 mM Tris, 150 mM NaCl, 0.1% Tween-20) for 2 h at room temperature. The membrane blot was probed with purified FtsZ1 anti-bodies with a dilution of 1:2500 for 1 h at room temperature, and then the secondary antibodies (Jiaxuan biotech) with a dilution of 1:10,000 for 1 h at room temperature. The signals were generated with an eECL Western Blot Kit (Beijing ComWin Biotech Company) and developed with a film.

### Yeast Two-Hybrid Analysis

Gene fragments of *CreARC6^148–627^*, *CreZ1*, *CreZ2*, *CreZ1^F473G^*, *CreZ2^F425G^* were amplified with the primers CreARC6-5 and CreARC6-2, CreZ1-8 and CreZ1-9, CreZ2-5 and CreZ2-6, CreZ1-10 and CreZ1-M1, CreZ2-9 and CreZ2-M1, respectively. *CreARC6^148–627^* was digested with EcoRⅠ and BamHⅠ and cloned into pGADT7. *CreZ1*, *CreZ2*, *CreZ1^F473G^*, *CreZ2^F425G^* were digested with NdeⅠ and EcoRⅠ and cloned into pGBKT7. Gene fragments *PpARC6^175–528^*, *PpZ1B* and *PpZ2-1* were amplified with primers PpARC6-1 and PpARC6-2, PpZ1B-9 and PpZ1B-11, PpZ2-1-3 and PpZ2-1-5, respectively. *PpARC6^175–528^* was digested with ClaⅠ and SacⅠ and cloned into pGADT7. PpZ1B and PpZ2-1 were digested with NdeⅠ and BamHⅠ, and cloned into pGBKT7. Constructs of AD and BD combination were co-transformed into yeast strain AH109, and then cultured with 2D synthetic dropout medium (−Leu/–Trp) and 3D selective medium (Leu/–Trp/–His) at 30 °C for 3 d. To analyze the intensity of protein interaction, a gradient dilution of 1, 10^−1^ and 10^−2^ was used in the experiment.

## Supplementary Material

msae145_Supplementary_Data

## Data Availability

The FtsZ protein sequences obtained in this study are available in the National Center for Biotechnology Information (NCBI), and the accession numbers are shown in the online supplementary materials, Supplementary Material online. Additional supplementary data, Supplementary Material online, including the FtsZ protein sequences used in this study, are available online.
