# Supplementary material for: Evolution and Functional Differentiation of the C-terminal Motifs of FtsZs During Plant Evolution
Source: Mol Biol Evol. 2024 Jul 15;41(7):msae145. doi: 10.1093/molbev/msae145 (PMC11285052; doi:10.1093/molbev/msae145)
Supplement: msae145_Supplementary_Data [file msae145_supplementary_data.zip › Supplementary Materials - 24-6-17 - changes.pdf]

Supplementary Materials

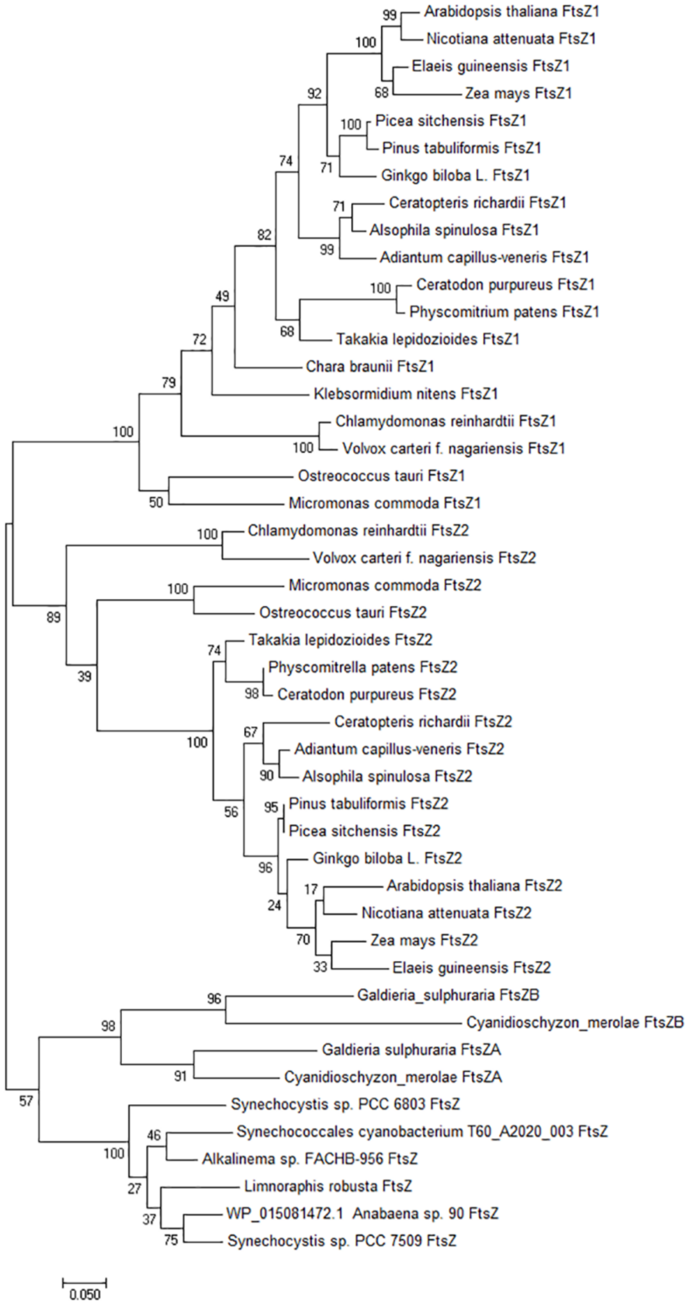

Figure. S1 A phylogenetic tree of the evolution relationship of FtsZ1and FtsZ2 in plants, and their homologous FtsZ in Cyanobacteria, and FtsZA and FtsZB in red algae, constructed using the maximum likelihood method. Bootstrap values, based on 1000 replicates, are displayed at corresponding nodes. The scale bar represents 0.05 amino acid substitutions per position.

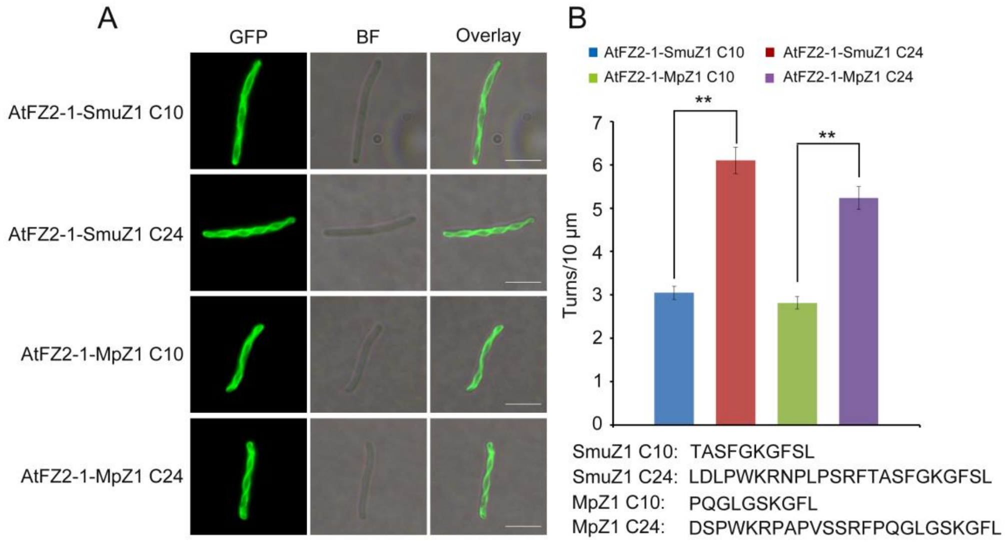

Figure. S2 Analysis of the membrane-binding ability of Zygnematophyta and liverwort FtsZ1 C-terminal sequences. (A) GFP-AtFZ2-1 with a fusion of SmuZ1 C10, SmuZ1 C24, MpZ1 C10 and MpZ1 C24 formed helical structures in *E. coli*. Bars = 5 μm. SmuZ1 C10, the last 10 amino acids of SmuZ1. SmuZ1 C24, the last 24 amino acids of SmuZ1. MpZ1 C10, the last 10 amino acids of MpZ1. MpZ1 C24, the last 24 amino acids of MpZ1. (B) Statistical analysis of the helical density of the FtsZ fusion proteins in (A). *t* test, ***P* < 0.01. Error bars represent the mean ±SD.

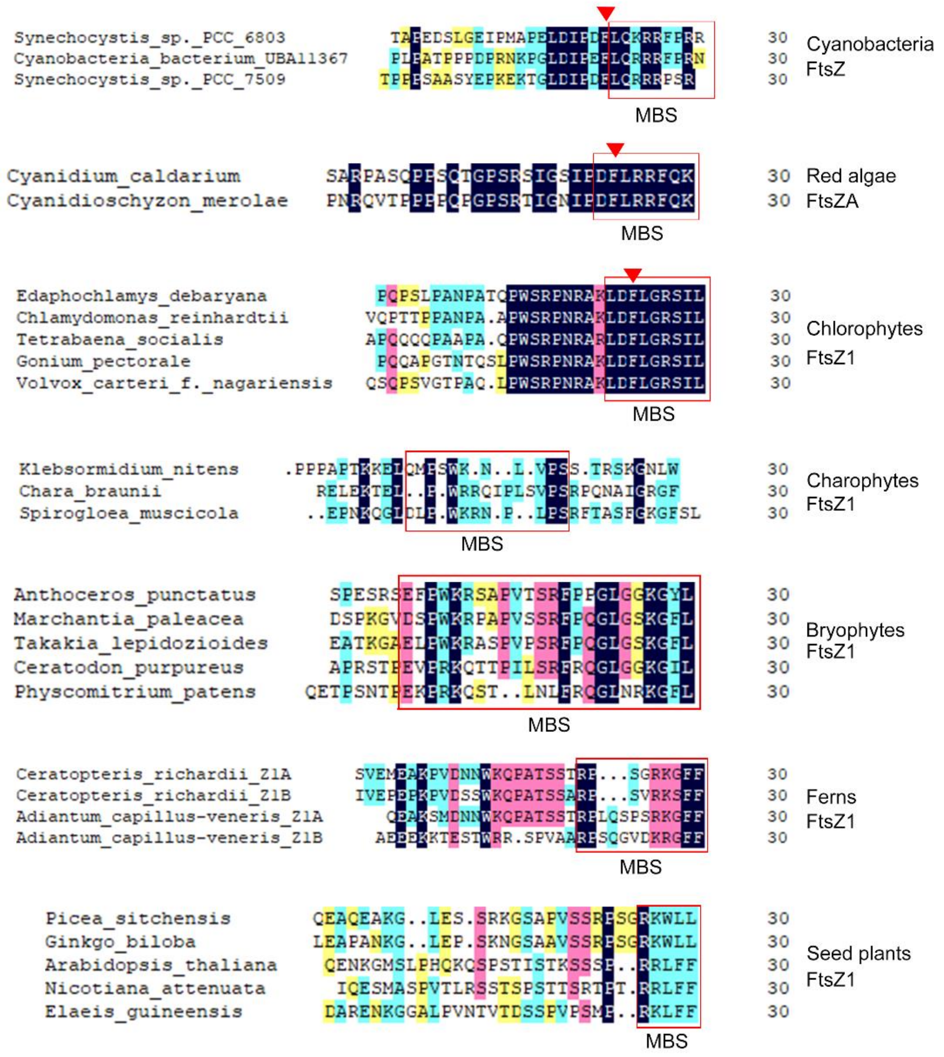

Figure. S3 Membrane-binding sequence (MBS) alignment of cyanobacteria FtsZ, red algae FtsZA and FtsZ1 in different species. Red boxes highlight the specific membrane-binding amino acid sequences (MBS), while red triangles indicate the key amino acid phenylalanine (F) known to be important for the interaction with Ftn2 or ARC6 (Maple, et al. 2005; Zhang, et al. 2016).

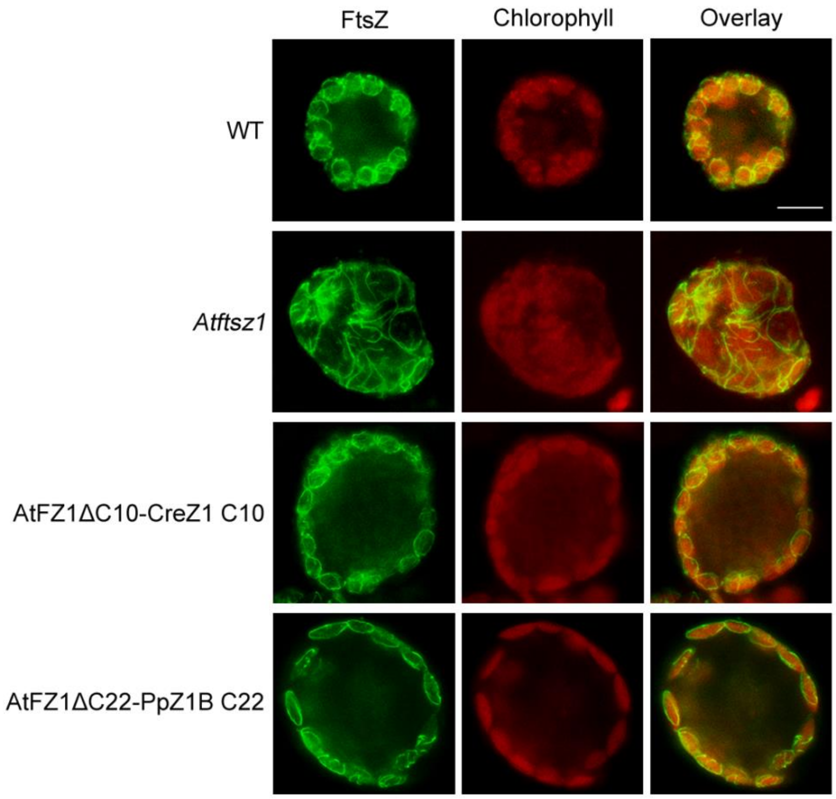

Figure. S4 Immunofluorescence staining of WT, *Atftsz1* and transgenic plants *AtFZ1ΔC10-CreZ1 C10*/ *Atftsz1* and *AtFZ1ΔC22-PpZ1B C22*/ *Atftsz1*. Bar = 10 μm. The bar refers to all images shown here.

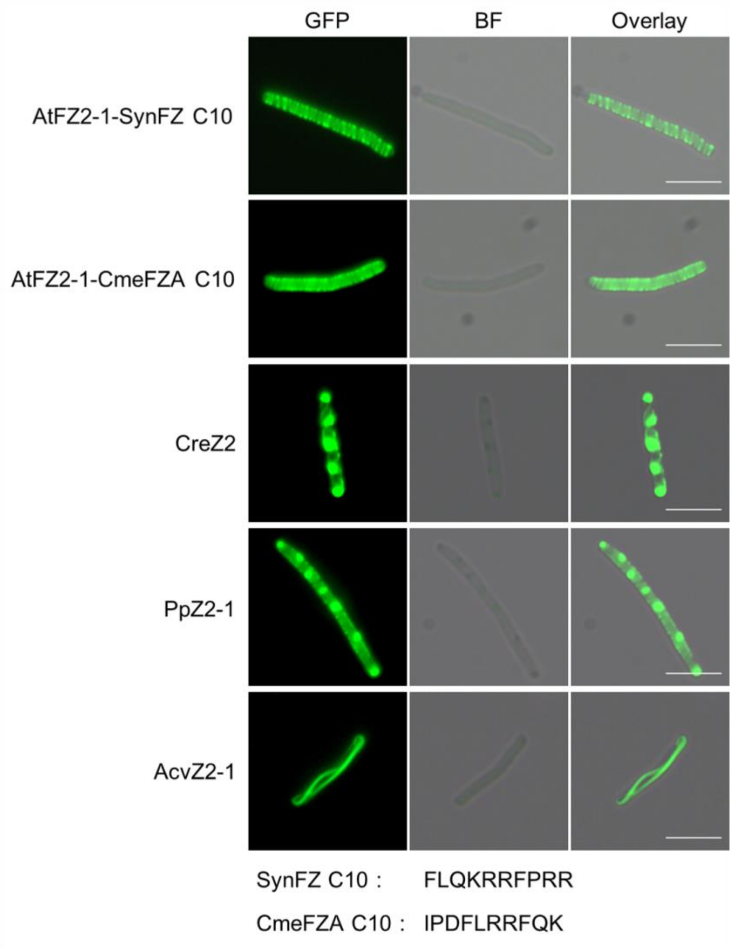

Figure. S5 SynFZ C10, CmeFZA C10, CreZ2 and PpZ2-1 exhibit membrane-binding activity, while AcvZ2-1 lacks this activity in *E. coli*. SynFZ C10 and CmeFZA C10 were fused to C-terminus of GFP-AtFZ2-1 and the fusion proteins formed dense helical structures in *E. coli*. SynFZ C10, the last 10 amino acids of SynFZ. CmeFZA C10, the last 10 amino acids of CmeFZA. CreZ2, PpZ2-1 and AcvZ2-1 were fused to the C-terminus of GFP and expressed in *E. coli*. Scale bars = 5 μm.

Supplemental Table S1. Amino acid sequences of FtsZ motifs in this study. Red fonts indicate mutant amino acid residues.

FtsZ motifs	Sequences of FtsZ motifs
CreZ1 C10	KLDFLGRSIL
CreZ1 C18	PWSRPNRAKLDFLGRSIL
KniZ1 C10	SSTRSKGNLW
KniZ1 C24	KKELQMPSWKNLVPSSTRSKGNLW
CbrZ1 C10	RPQNAIGRGF
CbrZ1 C24	ELPWRRQIPLSVPSRPQNAIGRGF
SmuZ1 C10	TASFGKGFSL
SmuZ1 C24	LDLPWKRNPLPSRFTASFGKGFSL
ApuZ1 C10	PPGLGGKGYL
ApuZ1 C24	EFPWKRSAPVTSRFPPGLGGKGYL
MpZ1 C10	PQGLGSKGFL
MpZ1 C24	DSPWKRPAPVSSRFPQGLGSKGFL
PpZ1B C10	RQGLNRKGFL
PpZ1B C10 ^{M1}	RQGLNRKGFF
PpZ1B C10 ^{M2}	RQGLNRKLFF
PpZ1B C22	EKPRKQSTLNLFRQGLNRKGFL
CpuZ1 C10	RQGLGGKGIL
CpuZ1 C24	EVPRKQTTPILSRFRQGLGGKGIL
DcoZ1 C10	SQGSNGKGYL
DcoZ1 C24	ELPWKRAAPVASRFSQGSNGKGYL
AcvZ1A C10	LQSPSRKGFF
AcvZ1A C10 ^{M1}	LQSPSRKLFF
AcvZ1A C24	DNNWKQPATSSTRPLQSPSRKGFF
CriZ1A C10	ARPSVRKSFF
CriZ1A C24	KPVDSSWKQPATSSARPSVRKSFF
PsZ1 C10	SRPSGRKWLL
AtFZ1 C10	KSSSPRRLFF
AtFZ1 C10 ^{M1}	KSSSPRRGFF
AtFZ1 C10 ^{M2}	KSSSPRRGFL
SynFZ C10	FLQKRRFPRR
CmeFZA C10	IPDFLRRFQK

Supplemental Table S2. The accession numbers of FtsZs in this study.

Groups	Species and proteins	Accession numbers
Cyanobacteria	*Synechocystis sp. PCC 6803* FtsZ	WP_010872126.1
	Anabaena sp. 90 FtsZ	WP_015081472.1
	Limnoraphis robusta FtsZ	WP_046280017.1
	Synechococcales cyanobacterium T60_A2020_003 FtsZ	MBF2079305.1
	Alkalinema sp. FACHB-956 FtsZ	WP_199306980.1
	Synechocystis sp. PCC 7509 FtsZ	WP_227501565.1
Rhodophytes	*Galdieria sulphuraria* FtsZA	XP_005707174.1
	Galdieria_sulphuraria FtsZB	EME30778
	Cyanidioschyzon_merolae FtsZA	XP_005538694.1
	Cyanidioschyzon_merolae FtsZB	XP_005537502.1
Chlorophytes	*Ostreococcus tauri* FtsZ1	XP_003080788.2
	Chlamydomonas reinhardtii FtsZ1	XP_001702420.1
	Micromonas commode FtsZ1	XP_002501749.1
	Volvox carteri f. nagariensis FtsZ1	XP_002955661.1
	Micromonas commode FtsZ2	XP_002499616.1
	Ostreococcus tauri FtsZ2	XP_003080256.1
	Chlamydomonas reinhardtii FtsZ2	AAM22891.1
	Volvox carteri f. nagariensis FtsZ2	XP_002952497.1
Charophytes	*Klebsormidium nitens* FtsZ1	GAQ85535.1
	Chara braunii FtsZ1	GBG61650.1
Bryophytes	*Marchantia paleacea* FtsZ1	KAG6549690.1
	Marchantia polymorpha subsp. ruderalis FtsZ1	BBM96926.1
	Ceratodon purpureus FtsZ1	KAG0556253.1
	Physcomitrium patens FtsZ1A	XP_024361262.1
	Physcomitrium patens FtsZ1B	XP_024403459.1
	Marchantia paleacea FtsZ2	KAG6545957.1
	Marchantia polymorpha subsp. ruderalis FtsZ2	BBN11270.1
	Ceratodon purpureus FtsZ2	KAG0584853.1
	Physcomitrium patens FtsZ2-1	XP_001765953.1
	Physcomitrium patens FtsZ2-2	CAB76386.1
Lycophytes	*Diphasiastrum complanatum* FtsZ1	KAJ7570013.1
	Diphasiastrum complanatum FtsZ2	KAJ7563863.1

Pteridophytes	*Ceratopteris richardii* FtsZ1A	KAH7373490.1
	Ceratopteris richardii FtsZ1B	KAH7294588.1
	Adiantum capillus-veneris FtsZ1A	MBC9855054.1
	Adiantum capillus-veneris FtsZ1B	MBC9836049.1
	Adiantum capillus-veneris FtsZ2-1	MBC9826855.1
	Adiantum capillus-veneris FtsZ2-2	MBC9829030.1
	Ceratopteris richardii FtsZ2-1	KAH7287669.1
	Ceratopteris richardii FtsZ2-2	KAH7439622.1
Gymnosperms	*Picea sitchensis* FtsZ1	ABK24653.1
	Picea sitchensis FtsZ2	ABR17393.1
Angiosperms	*Musa acuminata subsp. malaccensis* FtsZ1	XP_009407904.1
	Arabidopsis thaliana FtsZ1	NP_200339.1
	Cucumis sativus FtsZ1	XP_004149587.1
	Nicotiana attenuata FtsZ1	XP_019226802.1
	Dioscorea alata FtsZ1	KAH7660303.1
	Populus trichocarpa FtsZ1	XP_002300342.2
	Elaeis guineensis FtsZ1	XP_010913967.1
	Zea mays FtsZ1	ACG36412.1
	Musa acuminata subsp. malaccensis FtsZ2	XP_009408276.1
	Arabidopsis thaliana FtsZ2	NP_001323530.1
	Dioscorea alata FtsZ2	KAH7685671.1
	Elaeis guineensis FtsZ2	XP_010905974.1
	Zea mays FtsZ2	XP_008663752.1
	Cucumis sativus FtsZ2	XP_004143321.1
	Nicotiana attenuata FtsZ2	XP_019238887.1
	Populus trichocarpa FtsZ2	XP_024460336.1

Supplemental Table S3. Primers used in this study.

Primers	Sequences
NcoIGFP	5'-CTCCCATGGCAAGTAAAGGAGAAGAACTTTTCAC-3'
GFPEoRI	5'-CCAGAATTCACCACCTTTGTATAGTTCATCCATGCC-3'
AtFZ2-1-43	5'-CCTGAATTCGCCGCTCAGAAATCTGAATCTTC-3'
AtFZ2-1-44	5'-CCTAAGCTTTTAGACTCGGGGATAACGAGAGC-3'
AtFZ2-1-46	5'- CCAGAATTCGGCAGCTCTCGTTATCCC -3'
T7ter	5'- CCGCTGAGCAATAACTAGC-3'
CreZ1-1	5'-<br>CCTCTCGAGTCACAGAATGCTGCGGCCCAGAAAGTCCAGCT<br>TAAGCTTGACTCGGGGAT-3'
KniZ1-1	5'-<br>CCTCTCGAGCTACCACAGGTTTCCCTTCGATCGCGTCGAGGA<br>AAGCTTGACTCGGGGAT-3'
CbrZ1-1	5'-<br>CCTCTCGAGTCAGAATCCACGACCAATTGCGTTCTGAGGTCT<br>AAGCTTGACTCGGGGAT-3'
SmuZ1-1	5'-<br>CCTCTCGAGTCAGAGTGAGAATCCTTTCCCAAAGCTGGCAG<br>TAAGCTTGACTCGGGGAT-3'
ApuZ1-1	5'-<br>CCTCTCGAGTCAAAGATACCCTTTACCGCCAAGACCAGGAG<br>GAAGCTTGACTCGGGGAT-3'
MpZ1-1	5'-<br>CCTCTCGAGCTACAAAAAACCTTTGCTACCCAGACCCTGCG<br>GAAGCTTGACTCGGGGATA-3'
CpuZ1-3	5'-<br>CCTCTCGAGTTAGAGGATTCCCTTACCTCCGAGGCCCTGACG<br>AAGCTTGACTCGGGGAT-3'
SynFZ-1	5'-<br>CCTCTCGAGTTAACGACGCGGGAATCGACGTTTCTGCAAGA<br>AAAGCTTGACTCGGGGAT-3'
CmeFZA-1	5'-<br>CCTCTCGAGTTACTTTTGGAATCGACGCAGAAAATCCGGAAT<br>AAGCTTGACTCGGGGAT-3'
PpZ1B-1	5'-<br>CCTCTCGAGCTACAAAAACCCCTTTCGGTTAAGACCTTGGC<br>G AAGCTTGACTCGGGGAT-3'
DcoZ1-1	5'-<br>CCTCTCGAGTTACAGATAGCCCTTACCGTTTGAACCTTGAGA<br>AAGCTTGACTCGGGGAT-3'
AcvZ1A-1	5'-<br>CCTCTCGAGCTAGAAGAAACCTTTACGGCTCGGGCTCTGCA

	GAAGCTTGACTCGGGGATA-3'
CriZ1A-1	5'-CCTCTCGAGTCAGAAGAACGACTTCCTAACGCTCGGCCTTGCAAGCTTGACTCGGGGAT-3'
PsZ1-1	5'-CCTCTCGAGTCATAAAAGCCACTTCCTACCAGAAGGCCTAGAAAGCTTGACTCGGGGATA-3'
AtFZ1-46	5'-CCTCTCGAGCTAGAAGAAAAGTCTACGGGGAGAAGACGATTTAAGCTTGACTCGGGGATA-3'
PpZ1B-M1	5'-CCTCTCGAGCTAGAAAAACCCCTTTCGGTTAAG-3'
PpZ1B-M2	5'-CCTCTCGAGCTAGAAAAACAGCTTTCGGTTAAG-3'
AcvZ1A-M1	5'-CCTCTCGAGCTAGAAGAAAAGTTTACGGCTCG-3'
AtFZ1C-M1	5'-CCTCTCGAGCTAGAAGAAACCTCTACGGGGA-3'
AtFZ1C-M2	5'-CCTCTCGAGCTACAAGAAACCTCTACGGGGA-3'
CreZ1-2	5'-GTCCAGCTTGGCACGGTTGGGGCGGCTCCAGGGAAGCTTGACTCGGGGAT-3'
KniZ1-2	5'-GGGCACCAAGTTCTTCCAGCTCGGCATCTGAAGCTCCTTCTTAAGCTTGACTCGGGGAT-3'
CbrZ1-2	5'-TGATGGTACGCTCAGAGGGATTTGTCGACGCCAGGGTAGCTCAAGCTTGACTCGGGGAT-3'
SmuZ1-2	5'-GAACCGGGATGGTAGAGGATTGCGCTTCCAAGGCAAGTCCAGAAGCTTGACTCGGGGAT-3'
ApuZ1-2	5'-GAACCTGGACGTCACAGGGGCACTTCGCTTCCATGGAAATTCAAGCTTGACTCGGGGAT-3'
MpZ1-2	5'-AATCGGGACGAGACAGGTGCAGGTCTTTTCCAAGGGGAATCAAGCTTGACTCGGGGATA-3'
DcoZ1-2	5'-GAACCGTGATGCGACAGGTGCAGCTCTCTTCCAAGGCAGTTCAAGCTTGACTCGGGGAT-3'
CreZ2-7	5'-CCGTCGACGTCGCAGGAAGGCCGGAATAAGCTTGACTCGGGG AT-3'
PpZ1B-2	5'-TGGCGAAACAAGTTGAGAGTTGATTGCTTCCTAGGTTTCTCA

	AGCTTGACTCGGGGAT-3'
CpuZ1-1	5'-GAACCTCGACAGGATCGGCGTAGTCTGCTTTCTAGGCACCTCAAGCTTGACTCGGGGAT-3'
PpZ2-1-1	5'-GCCTCGCTTCCTTAAGAAACTTGGTATAAGCTTGACTCGGGGAT-3'
AcvZ1A-2	5'-GGTCTCGTGCTGCTAGTTGCCGGCTGCTTCCAATTGTTGTCAAGCTTGACTCGGGGATA-3'
CriZ1A-2	5'-ACTGCTAGTTGCAGGCTGCTTCCACGATGAGTCTACAGGCTTAAGCTTGACTCGGGGAT-3'
CreZ1-3	5'-CCTCTCGAGTCACAGAATGCTGCGGCCCAGAAAGTCCAGCTTGGCACGGT-3'
KniZ1-3	5'-CCTCTCGAGCTACCACAGATTTCCTTTCGATCGCGTAGAGGAGGGCACCAAGTTCTTCC-3'
CbrZ1-3	5'-CCTCTCGAGTCAGAATCCACGACCAATTGCGTTCTGAGGTCTTGATGGTACGCTCAGAG-3'
SmuZ1-3	5'-CCTCTCGAGTCAGAGTGAGAATCCTTTCCCAAAGCTGGCAGTGAACCGGGATGGTAGAG-3'
ApuZ1-3	5'-CCTCTCGAGTCAAAGATACCCTTTACCGCCAAGACCTGGAGGGAACCTGGACGTCACAG-3'
MpZ1-3	5'-CCTCTCGAGCTACAAGAAACCTTTGCTTCCCAGACCCTGAGGGAATCGGGACGAGACAG -3'
DcoZ1-3	5'-CGTCTCGAGTTACAGATAGCCCTTACCGTTTGAACCTTGAGAGAACCGTGATGCGACAG-3'
CreZ2-8	5'-CCTCTCGAGTTACTTGCCCTGCACCCGTCGACGTCGCAGGAA -3'
PpZ1B-3	5'-CCTCTCGAGCTACAAAAACCCCTTTCGGTTAAGACCTTGGCGAAACAAGTTGAGAGTTG-3'
CpuZ1-2	5'-CCTCTCGAGTTAGAGGATTCCCTTACCTCCGAGGCCCTGACGGAACCTCGACAGGATCG-3'
PpZ2-1-2	5'-

	CCTCTCGAGTTAATGACGTGTCTGGCCTCGCTTCCTTAAGAA-3'
AcvZ1A-3	5'-CCTCTCGAGCTAGAAGAAACCTTTACGGCTCGGGCTCTGCAGAGGTCTCGTGCTGCTAG-3'
CriZ1A-3	5'-CCTCTCGAGTCAGAAGAACGACTTCCTAACGCTCGGCCTTGCACTGCTAGTTGCAGGCT-3'
AtFZ1-10	5'-CCTGGATCCGACCCGTTATTGTTTCCTCTCC-3'
CreZ1-11	5'-GCCCAGAAAGTCCAGCTTGGTAGAGATAGTTGATGGAGAC-3'
PpZ1B-4	5'-TGGCGAAACAAGTTGAGAGTTGATTGCTTCCTAGGTTTCTCCAGAGACATGCCCTTGTT-3'
CreZ1-12	5'-CCTCCATGGTCACAGAATGCTGCGGCCCAGAAAGTCCAGCTT-3'
PpZ1B-5	5'-CCTCCATGGCTACAAAAACCCCTTTCGGTTAAGACCTTGGCGAAACAAGTTGAGAGTTG-3
CreARC6-5	5'-CCTGAATTCAAGCTGGCCGCTGGTCAC-3'
CreARC6-2	5'-CCTGGATCCTCAGGCCAGCACCTGCTCCA-3'
CreZ1-8	5'-CCTCATATGGTCTTCCGCCAGCACC-3'
CreZ1-9	5'-CCTGAATTCTCATAGAATGCTGCGGCC-3'
CreZ2-1	5'-CCAGAATTCATGGCCACTTTGTCTGTTC-3'
CreZ2-2	5'-CCTCTCGAGTTACTTGCCCTGCACC-3'
CreZ2-5	5'-CCACATATGATGGCCACTTTGTCTGTTC-3'
CreZ2-6	5'-CCTGAATTCTTACTTGCCCTGCACC-3'
CreZ1-10	5'-GGCCGCAGCATTCTGTGA-3'
CreZ1-M1	5'-CAGACCGTCCAGCTTGGCACG-3'
CreZ2-9	5'-TCCACACCAGAGGAAGACGCGCCA-3
CreZ2-M1	5'-GATTCCGGCCGGCCTGCGCC-3'
PpARC6-1	5'-CCTATCGATGGCCTGGCCGAAGATGAAGC-3'
PpARC6-2	5'-CCTGAGCTCTCAAGCCATGTGCGACCCATTG-3'
PpZ1B-9	5'-CCTCATATGGCACGGTCTGTGTATCCCA-3'
PpZ1B-11	5'-CCTGGATCCCTACAAAAACCCCTTTCGG-3'
PpZ2-1-3	5'-CCTCATATGATGCATTCTAGGTCAAGCG-3'
PpZ2-1-4	5'-CCTGGTACCATGCATTCTAGGTCAAGCG -3'
PpZ2-1-5	5'-CCTGGATCCTTAATGACGTGTCTGGCCTC-3'
AcvZ2-1-3	5'-CCTGAATTCCACATGCATTGTCCAAGGTTATC-3'
AcvZ2-1-6	5'-GCCAAGATAAAGGTTGTTGG-3'

Supplementary references

Maple J, Aldridge C, Møller SG. 2005. Plastid division is mediated by combinatorial assembly of plastid division proteins. *Plant J*. 43: 811-823.

Zhang M, Chen C, Froehlich JE, TerBush AD, Osteryoung KW. 2016. Roles of Arabidopsis PARC6 in Coordination of the Chloroplast Division Complex and Negative Regulation of FtsZ Assembly. *Plant Physiol*. 170: 250-262.
